# Cellular and Behavioral Characterization of *Pcdh19* Mutant Mice: subtle Molecular Changes, Increased Exploratory Behavior and an Impact of Social Environment

**DOI:** 10.1523/ENEURO.0510-20.2021

**Published:** 2021-08-10

**Authors:** Natalia Galindo-Riera, Sylvia Adriana Newbold, Monika Sledziowska, Cristina Llinares-Benadero, Jessica Griffiths, Erik Mire, Isabel Martinez-Garay

**Affiliations:** 1 Division of Neuroscience, School of Biosciences, Cardiff University, Cardiff CF10 3AX, United Kingdom; 2 Hodge Centre for Neuropsychiatric Immunology, Neuroscience and Mental Health Research Institute, Division of Psychological Medicine and Clinical Neurosciences, School of Medicine, Cardiff University, Cardiff CF24 4HQ, United Kingdom

**Keywords:** cortical lamination, impact of mutant littermates, neuronal subtypes, open field, single-cell RNAseq

## Abstract

Mutations in the X-linked cell adhesion protein PCDH19 lead to seizures, cognitive impairment, and other behavioral comorbidities when present in a mosaic pattern. Neither the molecular mechanisms underpinning this disorder nor the function of PCDH19 itself are well understood. By combining RNA *in situ* hybridization with immunohistochemistry and analyzing single-cell RNA sequencing datasets, we reveal *Pcdh19* expression in cortical interneurons and provide a first account of the subtypes of neurons expressing *Pcdh19*/*PCDH19*, both in the mouse and the human cortex. Our quantitative analysis of the *Pcdh19* mutant mouse exposes subtle changes in cortical layer composition, with no major alterations of the main axonal tracts. In addition, *Pcdh19* mutant animals, particularly females, display preweaning behavioral changes, including reduced anxiety and increased exploratory behavior. Importantly, our experiments also reveal an effect of the social environment on the behavior of wild-type littermates of *Pcdh19* mutant mice, which show alterations when compared with wild-type animals not housed with mutants.

## Significance Statement

*PCDH19* mutations cause epileptic encephalopathy in humans, but the underlying pathophysiology is not completely understood. Here, we provide the first quantitative analysis of the cortical neuronal types expressing *Pcdh19* in the mouse and human neocortex, and of cortical layer composition in *Pcdh19* mutant animals, revealing the expression of *Pcdh19* in interneurons and the presence of small, but significant, changes in neuronal distribution. The findings of our behavioral analysis indicate not only reduced anxiety and increased exploratory behavior, but also an impact of the mutant genotype on the behavior of wild-type animals when housed in the same cage. This finding underscores the importance of selecting appropriate control cohorts to avoid missing relevant behavioral changes in mutant animals.

## Introduction

*PCDH19* is one of several genes located on the X chromosome known to impact neurodevelopment and behavior. Mutations in this gene were identified in patients with EIEE9 (Epileptic Encephalopathy, Early Infantile, 9; #300088, OMIM), also known as Girls Clustering Epilepsy (GCE), over a decade ago (Dibbens et al., 2008). Since then, over 140 mutations have been described (Kolc et al., 2019), consolidating *PCDH19* as the second most relevant gene in epilepsy after *SCNA1* (Depienne and Leguern, 2012; Duszyc et al., 2015). The pathogenicity of *PCDH19* mutations is dependent on cellular mosaicism, and therefore the disorder follows an unusual inheritance, manifesting in heterozygous (HET) females and in males with somatic mutations (Depienne et al., 2009; Terracciano et al., 2016). Symptoms develop in affected patients during early infancy, often within the first year of life, and display clustered seizures, varying degrees of cognitive impairment, and other comorbidities, including autism spectrum disorder (ASD), attention deficits, and obsessive-compulsive features (Kolc et al., 2020).

*PCDH19* codes for Protocadherin 19, a calcium-dependent cell–cell adhesion molecule of the cadherin superfamily. This δ2-protocadherin has six extracellular cadherin repeats, a single transmembrane domain, and a cytoplasmic tail with two conserved motives of unknown function (CM1 and CM2; Wolverton and Lalande, 2001). In addition, a WRC (WAVE regulatory complex) interacting receptor sequence (WIRS) downstream of CM2 allows PCDH19 to interact with the WAVE (WASP family Verprolin homolog) regulatory complex, enhancing its Rac1-mediated activation (Chen et al., 2014). PCDH19 is involved in different processes, ranging from neurulation and organization of the optic tectum in zebrafish (Emond et al., 2009; Cooper et al., 2015) to neurogenesis and regulation of GABAergic transmission in mammals (Fujitani et al., 2017; Bassani et al., 2018; Homan et al., 2018; Lv et al., 2019; Serratto et al., 2020). In addition, PCDH19 is involved in gene expression regulation with estrogen receptor α (Pham et al., 2017), and mutations in *PCDH19* lead to a deficiency of the neurosteroid allopregnanolone and of other neuroactive steroids (Tan et al., 2015; Trivisano et al., 2017). Two very recent publications have also addressed the role of PCDH19 in synapse formation in hippocampal cells (Hoshina et al., 2021; Mincheva-Tasheva et al., 2021).

To date, three different *Pcdh19* knock-out (KO) mouse models have been developed to explore the function of PCDH19. The first, produced by Taconic Biosciences, has the first three exons of the gene replaced by a β-galactosidase and neomycin (*LacZ-neo*) resistance cassette (Pederick et al., 2016). The second model retains exons 2 and 3, with a *LacZ-neo* selection cassette replacing exon 1, which encodes the entire extracellular and transmembrane domains (Hayashi et al., 2017). The third was created by CRISPR-Cas9-mediated deletion of exon 1 (Hoshina et al., 2021). Lack of *Pcdh19* mRNA and protein was confirmed for two of the models (Pederick et al., 2016; Hoshina et al., 2021), and no major anatomic defects were reported in any of the three mutant animal lines. However, increased neuronal migration has been described (Pederick et al., 2016), as well as behavioral alterations (Hayashi et al., 2017; Lim et al., 2019; Hoshina et al., 2021). In addition, heterozygous females display a striking segregation of *Pcdh19*-expressing and nonexpressing progenitors in the developing cortex and altered electrocorticogram traces (Pederick et al., 2018), as well as presynaptic defects in the hippocampal mossy fiber synapse that lead to long-term potentiation abolishment (Hoshina et al., 2021).

Although no major abnormalities in cortical architecture have been reported in either KO mouse model, no detailed quantitative analysis has been conducted yet. Similarly, while RNA *in situ* hybridization (ISH) revealed the strongest *Pcdh19* expression in layers II/III and V(a) in mice (Pederick et al., 2016; Hayashi et al., 2017), the neuronal subtypes expressing *Pcdh19* have not been characterized, possibly because of the difficulty of labeling PCDH19-expressing cells with current antibodies. Here we report on the identity of *Pcdh19*-expressing excitatory and inhibitory neurons in the mouse and human cortex, focusing mainly on somatosensory areas. We also uncover alterations in cortical neuronal distribution in the somatosensory cortex of the Taconic Biosciences *Pcdh19* mutant animals, as well as robust differences in the behavior of heterozygous females, including preweaning alterations and an impact of mutant animals on the behavior of their wild-type (WT) littermates.

## Materials and Methods

### Experimental animals

Animals were housed under a 12 h light/dark cycle with *ad libitum* access to water and food, and controlled temperature and humidity. All experiments using mice were approved by the local ethical boards and conducted following the directions of the UK Animal Scientific Procedures Act (update 1986).

C57BL6/J WT animals were purchased from Charles River Laboratories, and the *Pcdh19* KO line (TF2108) was acquired from Taconic Biosciences.

Experimental matings for anatomic and cellular characterization, as well as for behavioral analysis were set up using wild-type males and *Pcdh19* HET females to produce litters with WT males and females, KO males, and HET females.

### Analysis of single-cell RNA sequencing datasets

Gene expression matrices and metadata were downloaded from https://portal.brain-map.org/atlases-and-data/rnaseq. Analysis and visualization were conducted using R version 3.6.3, assisted by RStudio version 1.2.1335. Raw counts were normalized to account for library size (total sum of counts per cell) and transformed to counts per million (CPM) using R package scater version 1.16.2. Violin plots were generated with R packages gridExtra version 2.3 and ggplot2 version 3.3.1. River plots were made with R packages gridExtra version 2.3, ggplot2 version 3.3.1, and ggforce version 0.3.2.

### Tissue processing

Animals were perfused with PBS followed by 4% paraformaldehyde (PFA) in PBS. After perfusion, brains were extracted and postfixed in PFA 4% overnight at 4°C. For RNA ISH, brains were then cryoprotected in 30% sucrose in PBS before embedding in OCT compound (Tissue-Tek) before freezing. Samples were stored at −80°C until sectioning. Sections of 12 or 20 μm were cut with a cryostat (model CM3050, Leica Systems) and stored at −80°C until use. For immunostaining, fixed brains were briefly washed in PBS and embedded in 4% low-melting point agarose. Sections of 50 μm were cut with a vibrating microtome (model VT1000S, Leica Systems) and stored in PBS with 0.05% sodium azide at 4°C until use.

### RNA *in situ* hybridization and immunohistochemistry

The probe to detect *Pcdh19* has been described previously (Gaitan and Bouchard, 2006). Its sequence was amplified using primers Pcdh19e1-F, 5′-CACCAAGCAGAAGATTGACCGAG-3′, and Pcdh19e1-R, 5′-GCCTCCCATCCACAAGAATAGTG-3′, and cloned into pCRII-Blunt-TOPO (Thermo Fisher Scientific). This plasmid was then used to generate digoxigenin (DIG)-labeled sense and antisense probes.

Thawed sections were postfixed in 4% PFA, endogenous peroxidases were quenched with 3% hydrogen peroxidase, and slices were then acetylated in a 0.25% acetic anhydride solution. Prehybridization took place in prewarmed hybridization buffer (50% formamide, 0.1% Tween-20, 0.25% CHAPS, 250 μg/ml yeast tRNA, 500 μg/ml herring sperm, 5× Denhardt’s solution, 5× SSC, 50 μg/ml heparin, 2.5 mm EDTA) for 1 h at 65°C. Slices were hybridized with the denatured sense or antisense probes overnight at 65°C in a humidified chamber. The next day, slides were washed with 0.2× SSC (Thermo Fisher Scientific) and PBST, and then blocked in ISH blocking solution (10% Denhardt’s solution and 0.1% Triton X-100 in PBS) for 20 min at room temperature (RT). After blocking, brain slices were incubated in primary antibody for 1 h at RT, washed in PBST, and incubated in secondary antibody for 1 h at RT. The antibodies used are described below. Slides were then washed in PBST, equilibrated in TN buffer (150 mm NaCl and 100 mm Tris, pH 7.5 in water), and incubated for 30 min in 1:2000 HRP-coupled anti-DIG antibody (catalog #11207733910, Sigma-Aldrich). Following the incubation, tissue was rinsed in TNT (TN+ 0.5% Tween) and immersed in Cy3-Tyramide (TSATM Plus Cy3 Fluorescence Kit; catalog #NEL744001KT, Perkin-Elmer) in a 1:50 dilution dissolved in the amplification diluent. Slides were then washed, counterstained with DAPI, and mounted with DAKO Mounting Medium.

### Immunohistochemistry

Antigen retrieval was performed for staining with antibodies against RORB (RAR Related Orphan Receptor B), SATB2 (Special AT-Rich Sequence-Binding Protein 2), parvalbumin (Pvalb), and calretinin (CR), with the tissue either immersed in a 10 mm citrate buffer, pH 6, at 95°C for 5 min (RORB and SATB2) or 10 min (Pvalb, CR) before blocking. Coronal sections of 50 μm were blocked (4% BSA, 3% donkey serum, 0.1% Triton X-100 in PBS) at RT for 1 h. The tissue was then incubated in primary antibody diluted in blocking solution overnight at 4°C. Primary antibodies used for immunostaining were as follows: anti-CUX1 (Cut Like Homeobox 1) rabbit polyclonal (1:200; catalog #11733, Proteintech, or catalog #sc-13 024, Santa Cruz Biotechnology); anti-CTIP2 (COUP-TF-interacting protein 2) rat monoclonal (1:250; catalog #ab18465, Abcam); anti-SATB2 mouse monoclonal (1:400; catalog #ab51502, Abcam); anti-RORB rabbit polyclonal (1:200; catalog #17635-1AP, Proteintech); anti-TBR1 (T-Box Brain Transcription) rabbit polyclonal (1:350; catalog #ab31940, Abcam); anti-Pvalb rabbit polyclonal (1:10,000 or 1:500 for ISH; catalog #PV27, Swant); anti-CB rabbit polyclonal (1:5000; catalog #CB38, Swant); anti-CR mouse polyclonal (1:1000; catalog #AB5054, Merck); anti-SST (somatostatin) rat monoclonal (1:200; catalog #MAB354, Merck); anti-L1CAM (L1 Cell Adhesion Molecule) rat monoclonal (1:500; catalog #MAB5272, Merck); and anti-Neuropilin1 goat polyclonal (1:300; catalog #AF566, R&D Systems).

Slices were then rinsed in PBS and incubated with secondary antibodies coupled to fluorochromes (Alexa Fluor range, Thermo Fisher Scientific) for 1 h at RT. Nuclei were counterstained with DAPI for 10 min, washed again in PBS, and mounted with DAKO Mounting Medium.

### Image acquisition and analysis

Images were acquired using a confocal laser scanning microscope (Model LSM 780, Carl Zeiss) and ZEN Black software (version 2.0; Carl Zeiss). Image analysis was conducted with ImageJ Fiji software (Schindelin et al., 2012). For quantification, the cortical wall was divided into 10 horizontal bins of equal width. The number of marker-positive cells in each bin was quantified and is shown as the mean (±SEM) percentage relative to the total number of cells in all 10 bins.

### Behavioral analysis

Behavioral tests were conducted at postnatal day 21 (P21; preweaning) and in young adults (P60 and over). Two different WT controls were tested: WT littermates of the mutant animals [mixed-genotype housed (MGH) mice] and animals from pure WT litters [single-genotype housed (SGH) mice]. The WT parents of the SGH animals were derived from the *Pcdh19* colony. Mice were habituated to the new environment by taking them to the behavioral room 30 min before the tests. Mice were handled with open hands to reduce anxiety levels and a maximum of one behavioral test was performed per day.

### Open field

Open field behavioral analysis was performed on 2 consecutive days, using the first day to habituate the mice to the new environment. Mice were allowed to explore freely, in the dark, for 20 min, in an open field arena (40 × 40 cm). Spontaneous locomotion was recorded using a computer-linked video camera (The Imaging Source) located above the arena and an infrared illumination box (Tracksys) located underneath the arena. The EthoVision XT software (Noldus) was used to analyze the total distance traveled, the distance traveled in intervals of 5 min, and the time spent in the center of the arena. The center of the arena was defined as the area separated from the wall by ≥5 cm.

### Elevated plus maze

Each mouse was left to explore freely for 5 min in a maze consisting of the following four perpendicular arms (40 × 7 cm): two open arms (1 cm high) and two closed arms (16 cm high), in a well lit room. Behavior was recorded using a computer-linked video camera (The Imaging Source) located above the maze. The total time spent in the open arms was measured using EthoVision XT software (Noldus).

### Social interaction

At P21, test pups were habituated to the arena for 3 min. Subsequently, WT females in estrus, unfamiliar to the pups, were added to the cage, and both mice were allowed to interact with each other for another 3 min in a well lit room. The interaction between the pups and the females was recorded using a computer-linked video camera (The Imaging Source) located above the arena. Videos were manually scored, and interaction was recorded when both mice were within 2 cm of each other, not including tail–tail interactions.

At P60, only female mice were tested for social interaction. In this case the unknown WT females were not required to be in estrus.

To determine which females were in estrus, vaginal smears were stained with Giemsa solution (Polysciences; Caligioni, 2009) before the experiment.

### Twenty-four hour activity

P60 experimental mice were placed in individual clear boxes (40 × 24 × 18 cm) and allowed to roam free for 24 h with *ad libitum* access to food and water and their normal 12 h light/dark cycle. Three infrared beams traversed each cage at the bottom. Data were analyzed using the MED-PC IV software suite and extracted using the MPC2XL program. The number of beam breaks in 24 h and in 1 h slots, as well as the total number of beam breaks during the light and dark periods were analyzed.

### Experimental design and statistical analysis

For all experiments, individual animals were considered the experimental unit and the data obtained from each animal were averaged if more than one quantification was performed (e.g., when analyzing several brain slices from the same animal). Experimenters were blind to the genotype of the animals until all quantification or scoring was completed. Statistical analysis was performed using GraphPad Prism version 9 (cortical lamination analysis) or R version 3.6.2 (behavior; R Foundation for Statistical Computing). Normality of the data was tested using the Shapiro–Wilk test, and homogeneity of variance was assessed with Levene’s test. If either assumption was violated, an appropriate nonparametric test was used. Comparisons between two groups were performed using a two-tailed independent-samples *t* test for normal data, or a Mann–Whitney test if data distribution did not meet normality criteria. If the variance of the two groups differed, a Welch correction was applied. For comparison of more than two groups, ANOVA was used for normal data and a Kruskal–Wallis test if the assumption of normality was not met. If only the assumption of homogeneity of variance was not met, a Welch’s ANOVA was used. The *post hoc* test following ANOVA was adjusted according to Tukey’s test HSD or, in the case of the social interaction analysis, Dunnett’s test. A Kruskal–Wallis test was followed by Dunn’s correction, and Welch’s ANOVA was followed by Games–Howell correction. Statistical data are presented as the mean ± SEM for formal tests. To carry out estimation statistics for the behavioral experiments, data were introduced into the form available at www.estimationstats.com, in the section for multiple two-group arrangements to obtain the mean differences between groups and their corresponding 95% confidence intervals (CIs). The *y*-axis limits were set for optimal display of the raw data, and the graphs obtained were directly used in the figures of the article. Calculation of the unbiased Cohen’s *d* for each comparison, as well as its 95% CI, was conducted using the esci module on jamovi version 1.6 (The jamovi Project; https://www.jamovi.org).

## Results

### *Pcdh19* is expressed by different subtypes of cortical projection neurons and interneurons

**Figure 1.**
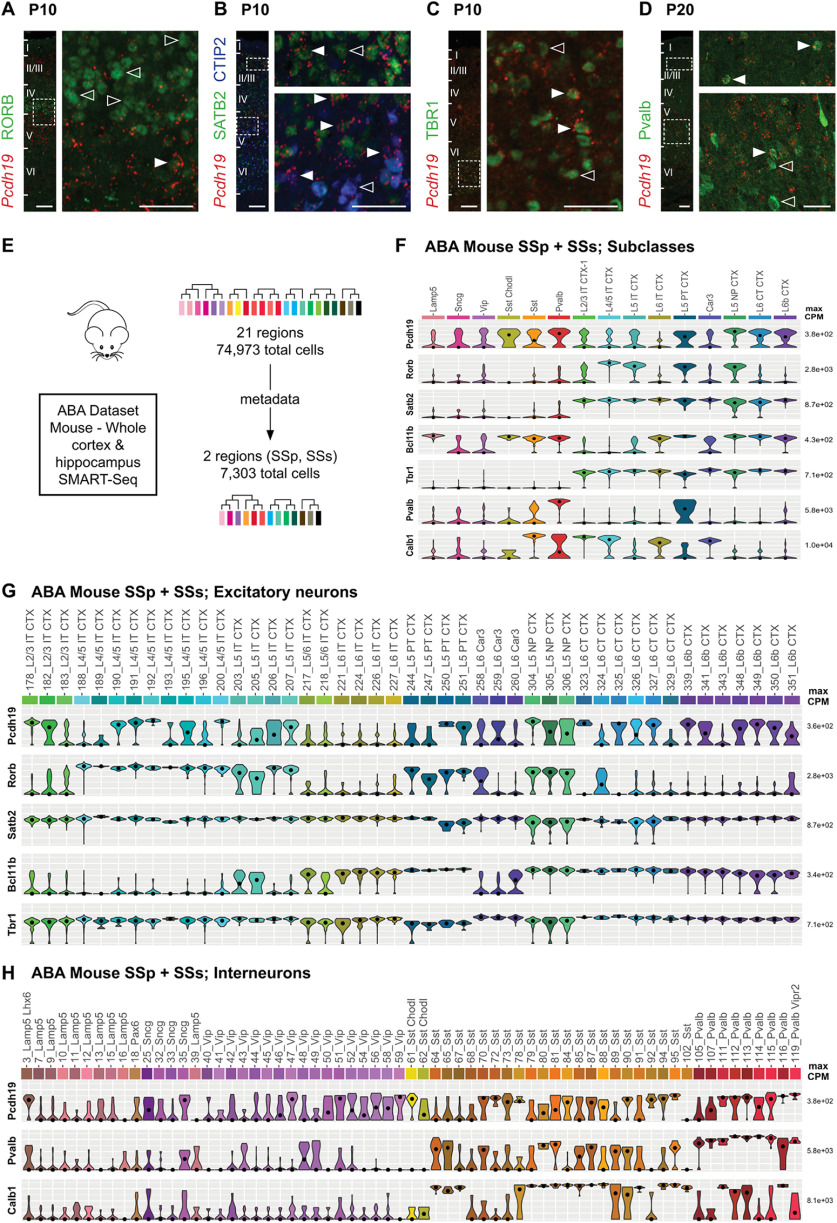
*Pcdh19* is expressed by excitatory and inhibitory neurons in the mouse cortex. ***A–D***, Confocal micrographs of P10 (***A–C***) and P20 (***D***) cortical slices hybridized with an RNA probe against *Pcdh19* (red) and antibodies against RORB (green; ***A***), SATB2 and CTIP2 (green and blue, respectively; ***B***), TBR1 (green; ***C***), and parvalbumin (Pvalb, green; ***D***). The left panel shows the entire cortical wall, with boxes indicating the regions enlarged in the right panels. White arrowheads point to double-positive cells, empty arrowheads point to single-positive cells (*Pcdh19* negative). Scale bars: left panels, 100 μm; right panels, 50 μm. ***E***, Strategy of the analysis of the mouse whole cortex and hippocampus dataset. ***F***, Violin plots representing gene expression and distribution for *Pcdh19* and the markers used in ***A–D*** in the 15 subclasses that the SSp and SSs neurons analyzed belong to. Four extra subclasses with five or fewer cells are not included in the figure. ***G***, ***H***, Violin plots representing gene expression and distribution for *Pcdh19* and the markers used in ***A–D*** in the different excitatory (***G***) and interneuronal (***H***) clusters defined in the study by Yao et al. (2020); Allen Brain Atlas, Whole Cortex & Hippocampus - SMART-SEQ (2019) with 10× Smart-Seq Taxonomy (2020). Dots indicate the median value of the cluster in CPM. CPM values are displayed on a log_10_ scale. For simplicity, clusters belonging to the four subclasses not included in ***F*** and any cluster with less than three neurons also are not represented in this figure. Gene expression and distribution of *Pcdh19* in cortical excitatory and inhibitory neurons of the Allen Brain Atlas Mouse Whole Cortex & Hippocampus dataset, both globally and by specific brain region, can be found in Extended Data Figures 1-1 and 1-2, respectively.

Previous RNA ISH studies have shown two main areas of *Pcdh19* expression in the adult cortex, corresponding to the upper regions of layer V (layer Va) and II/III (Hertel and Redies, 2011; Pederick et al., 2016). However, a detailed analysis of the cortical neuronal subtypes expressing *Pcdh19*, an important consideration given the cellular diversity of the cortex, is still lacking. To address this question, ISH against *Pcdh19* was combined with immunohistochemistry (IHC) against several cortical markers for principal neurons and interneurons in the somatosensory cortex at P10 and P20, respectively (Fig. 1*A–D*). At P10, *Pcdh19*^+^ cells were found to coexpress markers for layer IV neurons (RORB; Fig. 1*A*), callosal projection neurons (SATB2; Fig. 1*B*), corticospinal neurons (CTIP2; Fig. 1*B*), and corticothalamic neurons (TBR1; Fig. 1*C*). The strongest coexpression was seen in SATB2^+^ neurons, whereas RORB^+^ cells showed weaker expression and in a smaller proportion of cells. CTIP2^+^ neurons with strong *Pcdh19* expression tended to be located in the upper half of layer V, whereas TBR1^+^ cells coexpressing *Pcdh19* were generally located at lower levels. At P20, we identified interneurons coexpressing *Pcdh19* with parvalbumin in layers II/III and V (Fig. 1*D*), as well as double-positive cells for calbindin and *Pcdh19* (data not shown). These data suggest that in juvenile animals *Pcdh19* is expressed in both intratelencephalic and corticofugal projection neurons and reveal a previously unreported expression in subpopulations of cortical interneurons.

10.1523/ENEURO.0510-20.2021.f1-1 Figure 1-1 Gene expression and distribution of Pcdh19 in cortical excitatory projection neurons of the Allen Brain Atlas Mouse Whole Cortex & Hippocampus - SMART-SEQ (2019) with 10x Smart-Seq Taxonomy (2020), represented by violin plots. The first row shows the overall expression of Pcdh19 in the combined dataset excluding hippocampal regions for simplicity. Subsequent rows show expression by cortical region. Dots indicate the median value of the population. Absence of a violin plot in a row indicates that none or fewer than three cells from that particular cortical region were mapped to the corresponding neuronal cluster. Black and red lines indicate consistent low and high expression of Pcdh19 across areas, respectively; asterisks highlight clusters with marked variation in Pcdh19 expression across cortical regions. ACA, Anterior cingulate area; AI, agranular insular area; AUD, auditory areas; GU, gustatory areas; MOp, primary motor area; MOs-FRP, secondary motor area-frontal pole, cerebral cortex; ORB, orbital area; PL-ILA, prelimbic - infralimbic areas; PTLp, posterior parietal association areas; RSP, retrosplenial area; TEa-PERI-ECT, temporal association areas-perirhinal area-ectorhinal area; VIS, visual areas; VISp, primary visual area. Download Figure 1-1, TIF file.

**Figure 2.**
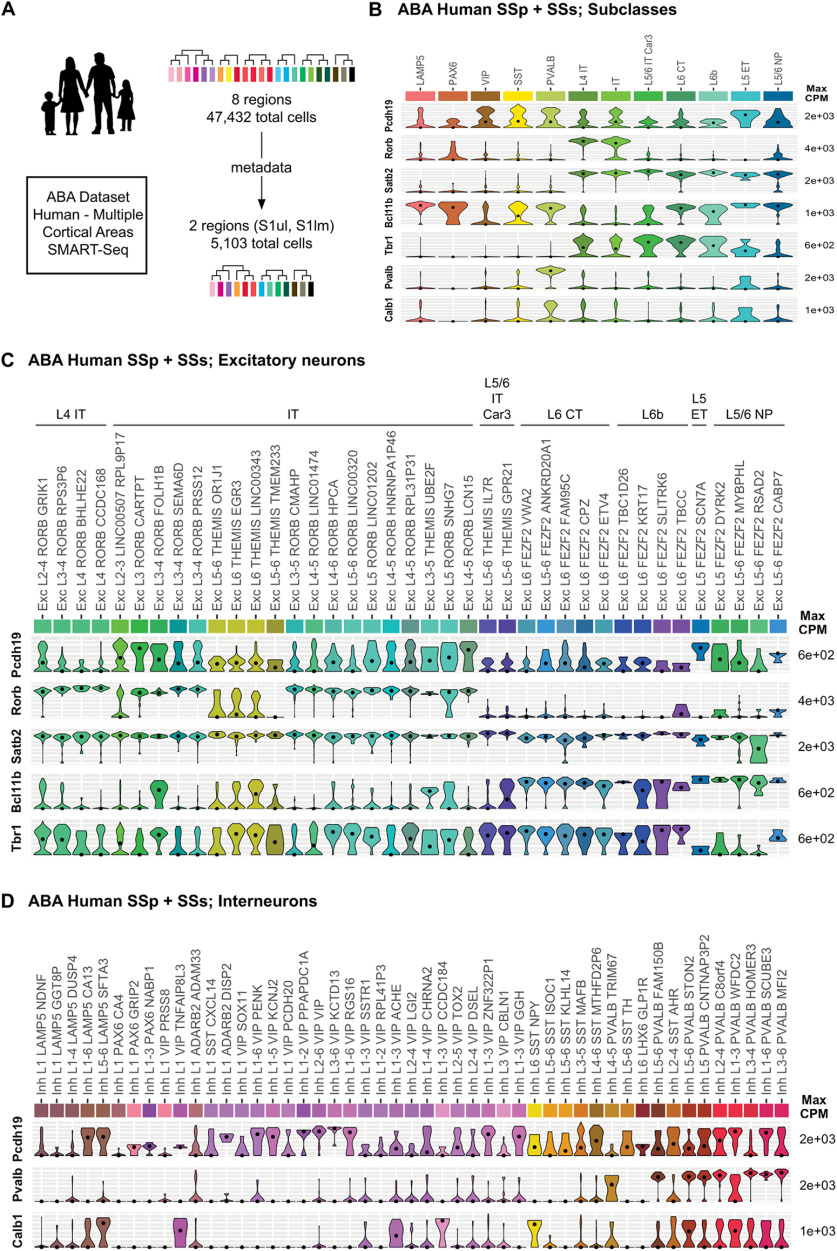
*PCDH19* is expressed by excitatory and inhibitory neurons in the human cortex. ***A***, Strategy of the analysis of the Human – Multiple Cortical Areas SMART Seq dataset. ***B***, Violin plots representing gene expression and distribution for *Pcdh19* and the markers used in ***A–D*** in the 12 subclasses that the sSC neurons analyzed belong to. ***C***, ***D***, Gene expression and distribution of *PCDH19* in the glutamatergic (***C***) and GABAergic (***D***) cell clusters of the human sSC, represented by violin plots. For the excitatory clusters, the corresponding subclasses are indicated at the top. Dots indicate the median value of the cluster in CPM. CPM values are displayed on a log_10_ scale. For simplicity, any cluster with less than three neurons is not represented in this figure. Gene expression and distribution of *PCDH19* in cortical excitatory and inhibitory neurons of the Allen Brain Atlas Human Multiple Cortical Areas dataset, both globally and by specific brain region, can be found in Extended Data Figures 2-1 and 2-2, respectively. For the strategy to indirectly correlate human and mouse clusters, the specific mouse and human neuronal GABAergic subtypes assigned to the different homology clusters and the correspondence between the nuclei from the MTG and the Multiple Cortical Areas datasets please see Extended Data Figure 2-3. IT (intratelencephalic), CT (corticothalamic), ET (extratelencephalic).

10.1523/ENEURO.0510-20.2021.f1-2 Figure 1-2 Gene expression and distribution of Pcdh19 in cortical inhibitory neurons of the Allen Brain Atlas Mouse Whole Cortex & Hippocampus - SMART-SEQ (2019) with 10x Smart-Seq Taxonomy (2020), represented by violin plots. The first row shows the overall expression of Pcdh19 in the combined dataset excluding hippocampal regions, for simplicity. Subsequent rows show expression by cortical region. Dots indicate the median value of the population. Absence of a violin plot in a row indicates that none or fewer than three cells from that particular cortical region were mapped to the corresponding neuronal cluster. Black and red lines indicate consistent low and high expression of Pcdh19 across areas, respectively; asterisks highlight clusters with marked variation in Pcdh19 expression across cortical regions. ACA, Anterior cingulate area; AI, agranular insular area; AUD, auditory areas; GU, gustatory areas; MOp, primary motor area; MOs-FRP, secondary motor area-frontal pole, cerebral cortex; ORB, orbital area; PL-ILA, prelimbic-infralimbic areas; PTLp, posterior parietal association areas; RSP, retrosplenial area; TEa-PERI-ECT, temporal association areas-perirhinal area-ectorhinal area; VIS, visual areas; VISp, primary visual area. Download Figure 1-2, TIF file.

The previous approach does not allow the identification of distinct molecular subtypes of excitatory and inhibitory neurons populating the neocortex. We thus turned to publicly available datasets of cortical single-cell RNA expression to ascribe molecular identities to *Pcdh19*-expressing neurons in the mouse adult somatosensory cortex. We chose the “Whole Cortex & Hippocampus - SMART-SEQ (2019) with 10×-Smart-Seq Taxonomy (2020)” dataset from the Allen Brain Atlas (available at https://portal.brain-map.org/atlases-and-data/rnaseq) that includes 76,307 single-cell transcriptomes with cluster-assigned identity isolated from a total of 21 adult cortical and hippocampal regions, including primary and secondary somatosensory cortex. The 74,973 cells for which metadata are available in this dataset are classified into 379 cell types, of which 236 are glutamatergic, 119 GABAergic, and 24 non-neuronal (Yao et al., 2020). We filtered for neurons originating from the primary somatosensory (SSp) and supplemental somatosensory (SSs) cortices using the dataset metadata, which yielded a total of 7303 neurons (Fig. 1*E*). Those neurons are assigned to 19 subclasses (Fig. 1*F*), although 4 of them contain <10 cells [Meis2 (5 cells), L2 IT RHP (4 cells), L5 IT TPE-ENT (3 cells), and L2/3 IT CTX-2 (2 cells)] and have not been included in Figure 1. Our analysis shows that, in agreement with our P10 and P20 results, *Pcdh19* expression is maintained in both excitatory and inhibitory populations in the adult somatosensory cortex that coexpress the markers of our ISH analysis (Fig. 1*E–H*).

In excitatory neurons of the adult somatosensory cortex, *Pcdh19* expression is lowest in the L6 IT CTX and L6 Car subclasses, where all clusters show consistent low median expression. However, in the remaining subclasses there is always at least one cluster that shows higher expression, indicating that there are *Pcdh19*-expressing neuronal populations in layers II/III and V, but also in layers VI and VIb, and possibly in layer IV, matching the results of our ISH analysis (Fig. 1*G*). The neurons expressing *Pcdh19* and SATB2 in layers II/III that we identified at P10 (Fig. 1*B*) could potentially represent clusters 178 and 182 of L2/3 intratelencephalically (IT) projecting neurons. In layer V, neurons expressing *Pcdh19* and CTIP2 may correspond to clusters 250 and 251, representing layer V neurons that project outside the cortex (PT), and/or clusters 304–306 of near-projecting neurons, whereas those expressing *Pcdh19* and SATB2, but not CTIP2, would be layer V IT neurons, matching those in clusters 190–192, 200, and 207. We also identified neurons expressing *Pcdh19* and TBR1 in layer VI (Fig. 1*C*) that could be corticothalamic neurons (clusters 323, 325, and 327) or layer VIb neurons (clusters 339 and 348–350).

A comparison between different brain regions (Extended Data Fig. 1-1) shows that, although expression levels in the different clusters are generally conserved across brain regions, there are also marked variations in several clusters that tend to manifest in just one or two specific regions.

As in the case of projection neurons, *Pcdh19* expression in interneurons of the adult somatosensory cortex is strongly cluster dependent. More specifically, the strongest average expression is found in the *Sst-Chodl* and *Pvalb* subclasses (Fig. 1*F*); however, there is considerable variation and several *Sncg*, *Vip*, and *Sst* clusters also express *Pcdh19* widely (Fig. 1*H*). To assign more meaningful identities to the interneuronal clusters expressing *Pcdh19*, we made use of the correlation provided between the GABAergic clusters generated from this dataset and the previous taxonomy from Tasic et al. (2018; Yao et al., 2020). *Sncg* neurons are *Vip*^+^, *Cck*^+^ multipolar or basket cells located mainly in upper layers, and two of their four subtypes have consistent *Pcdh19* expression. Three clusters of *Vip* interneurons also show relevant *Pcdh19* expression (Vip clusters 47, 51, and 59), with at least one of them corresponding to bipolar or multipolar cells (47_Vip). Within the *Pvalb* subclass, *Pcdh19* is expressed by chandelier cells (119_Pvalb Vipr2) and several subtypes of basket cells (Pvalb clusters 112–116). Finally, within the *Sst* subclass, *Pcdh19* expression is strongest in some subtypes of upper layer basket and Martinotti cells (Sst clusters 94 and 95), and in the long-range projecting population (61_Sst-Chodl). Again, variations in the level of *Pcdh19* expression within GABAergic clusters can be seen between brain regions (Extended Data Fig. 1-2), but, as was the case for excitatory neurons, differences tend to be limited to a few regions per cluster.

In summary, our analysis demonstrates that mouse *Pcdh19* expression is cluster specific in all glutamatergic and GABAergic subclasses in the somatosensory cortex and other cortical areas, being expressed by a heterogeneous neuronal population that includes discrete subtypes of cortical projection neurons and interneurons, with some variation between brain areas. Expression in non-neuronal cells is very low (data not shown).

### Human *PCDH19* is also expressed in excitatory and inhibitory neurons

Mutations in *PCDH19* cause severe impairments in brain function, yet the expression profile in human cortical neurons is unclear. We therefore extended our analysis to a publicly available human dataset from the Allen Brain Atlas (Human – Multiple Cortical Areas – SMART-seq; available at https://portal.brain-map.org/atlases-and-data/rnaseq), obtained from several brain areas (middle temporal gyrus, anterior cingulate gyrus, primary visual cortex, primary motor cortex, primary somatosensory cortex, and primary auditory cortex). This dataset comprises 49,417 cell nuclei (metadata available for 47,432 cell nuclei) and has allowed the definition of 56 excitatory and 54 inhibitory subtypes. We applied the same strategy as with the mouse dataset, filtering for those neurons originating in the somatosensory cortex, which reduced the dataset to 5103 neurons ascribed to 12 subclasses (Fig. 2*A*,*B*). Analysis of *PCDH19* expression in this restricted dataset revealed that, within glutamatergic neurons, *PCDH19* is primarily expressed in several excitatory neuronal subtypes, particularly Exc L5 FEZF2 SCN7A, which contains layer V neurons that project outside the cortex, and a series of clusters of intracortically projecting neurons spanning layers II–V, such as Exc L3 RORB CARTPT, Exc L3-4 RORB FOLH1B, Exc L5 RORB SNHG7, and Exc L4-5 RORB LCN15 (Fig. 2*C*). Low expression is evident in many other excitatory neurons of layers III–VI, although several layer IV and VI clusters tend to express much lower levels of *PCDH19*. A comparison between different brain regions beyond the somatosensory cortex (sSC) shows good correlation between the levels of *PCDH19* expression within clusters, with only a few exceptions (Extended Data Fig. 2-1). Regarding interneurons, *PCDH19* expression is highest in the L3-6 VIP KCTD13 subtype, with strong expression in most cells. In addition, *PCDH19* is also relatively highly expressed in several other VIP, LAMP5, SST, and PVALB subpopulations (Fig. 2*D*). A comparison between different brain regions reveals that, in general, *PCDH19* is expressed in each cluster at similar levels across areas. However, there are some exceptions, like L1 VIP PCDH20 interneurons, which show much higher *PCDH19* expression in the primary visual cortex (V1C) than in somatosensory areas [primary somatosensory cortex lower limb region (S1lm) and primary somatosensory cortex upper limb region (S1ul)] or L1-2 VIP RPL41P3, with higher *PCDH19* expression in motor areas (Extended Data Fig. 2-2).

10.1523/ENEURO.0510-20.2021.f2-1 Figure 2-1 Gene expression and distribution of PCDH19 in cortical excitatory projection neurons of the Allen Brain Atlas Human Multiple Cortical Areas dataset, represented by violin plots. The first row shows overall expression of PCDH19 in the combined dataset; subsequent rows show expression by brain region. Dots indicate the median value of the population. Absence of a violin plot in a row indicates that none or fewer than three cells from that particular brain region were mapped to the corresponding neuronal subtype. Black and red lines indicate consistent low and high expression of PCDH19 across areas, respectively; asterisks highlight clusters with marked variation in PCDH19 expression across cortical regions. CgG, Anterior cingulate gyrus; M1lm, primary motor cortex, lower limb region; M1ul primary motor cortex, upper limb region, A1C, primary auditory cortex. Download Figure 2-1, TIF file.

10.1523/ENEURO.0510-20.2021.f2-2 Figure 2-2 Gene expression and distribution of PCDH19 in cortical inhibitory neurons of the Allen Brain Atlas Human Multiple Cortical Areas dataset, represented by violin plots. The first row shows the overall expression of PCDH19 in the combined dataset; subsequent rows show expression by brain region. Dots indicate the median value of the population. Absence of a violin plot in a row indicates that none or fewer than three cells from that particular brain region were mapped to the corresponding neuronal subtype. Black and red lines indicate consistent low and high expression of PCDH19 across areas, respectively; asterisks highlight clusters with marked variation in PCDH19 expression across cortical regions. CgG, Anterior cingulate gyrus; M1lm, primary motor cortex, lower limb region; M1ul primary motor cortex, upper limb region, A1C, primary auditory cortex. Download Figure 2-2, TIF file.

**Figure 3.**
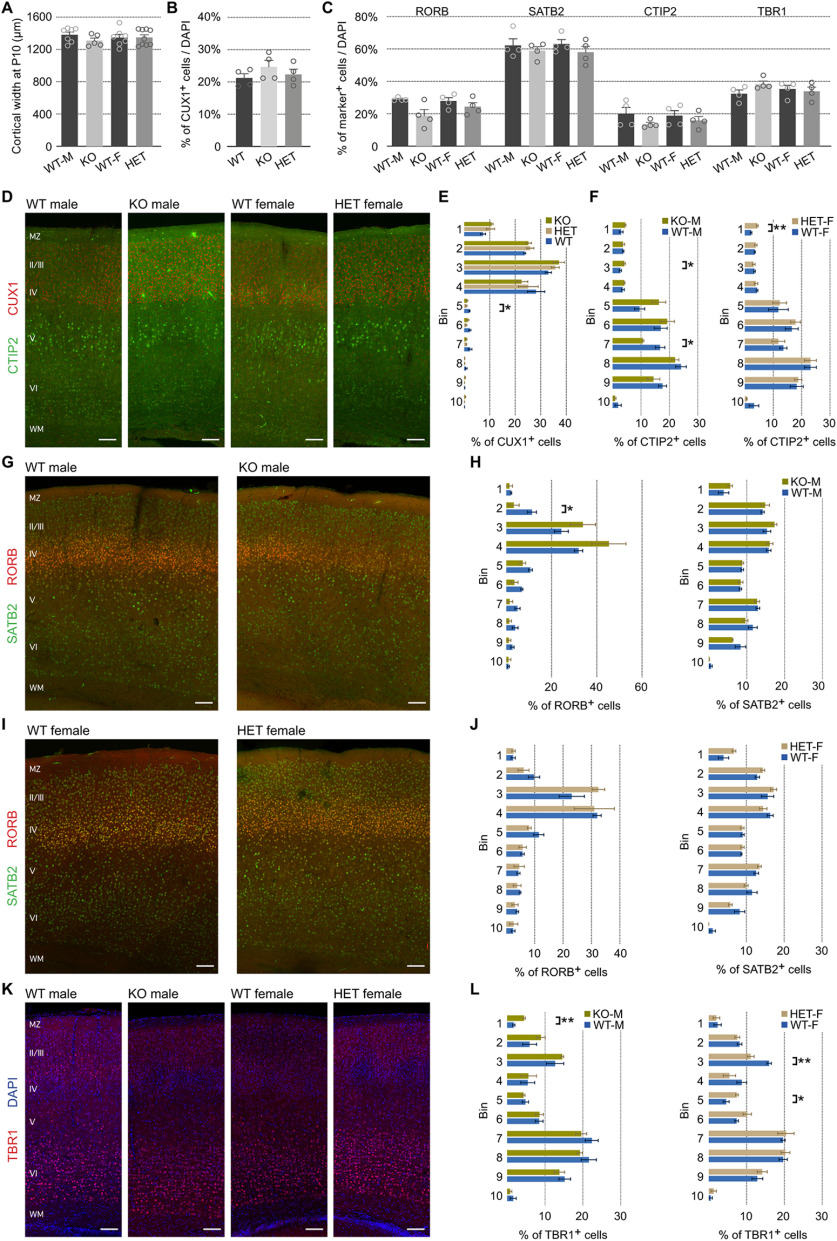
Subtle, but significant, changes in the distribution of cortical excitatory neurons in *Pcdh19* mutant animals. ***A***, Quantification of cortical width at P10 in *Pcdh19* WT and mutant animals, separated by sex. ***B***, Relative percentage of CUX1^+^ cells examined with respect to total DAPI^+^ cells in *Pcdh19* WT, HET, and KO animals. ***C***, Relative percentages of the different cortical markers examined with respect to total DAPI^+^ cells. Analysis performed separately for males and females. ***D***, Representative confocal micrographs of immunohistochemistry with anti-CUX1 (red) and anti-CTIP2 (green) antibodies on WT male, KO male, WT female, and HET female tissue. ***E***, Quantification of the percentage of CUX^+^ cells in each of 10 equal bins spanning the cortical wall. ***F***, Distribution of CTIP2^+^ cells in each of 10 equal bins spanning the cortical wall, shown as a percentage, for males (left) and females (right). ***G***, ***I***, Representative confocal micrographs of immunohistochemistry with anti-RORB (red) and anti-SATB2 (green) antibodies on WT and KO male tissue (***G***) and WT and HET female tissue (***I***). ***H***, ***J***, Quantification of the percentage of RORB^+^ (left) and SATB2^+^ (right) cells in each of 10 equal bins spanning the cortical wall. ***K***, Representative confocal micrographs of immunohistochemistry with anti-TBR1 (red) antibodies in WT male, KO male, WT female, and HET female tissue. Nuclei are counterstained with DAPI (blue). ***L***, Distribution of TBR1^+^ cells in each of 10 equal bins spanning the cortical wall, shown as a percentage for males (left) and females (right). All results are indicated as the mean ± SEM. A minimum of three images per brain, obtained from four animals originating from three different litters were analyzed for each condition. *p < 0.05; **p < 0.01. Scale bars, 200 μm.

10.1523/ENEURO.0510-20.2021.f2-3 Figure 2-3 ***A***, Diagram indicating the existing correlations between ABA mouse and human cortical datasets. ***B***, Diagram showing the homology clusters defined by Hodge et al. (2019) and the corresponding mouse and human neuronal subtypes assigned to each cluster for GABAergic neuronal clusters. ***C***, River plot showing the mapping of the nuclei from the MTG dataset to the subtypes defined by the Multiple Cortical Areas dataset, for inhibitory neurons. Download Figure 2-3, TIF file.

Having determined the levels of *Pcdh19/PCDH19* expression in the different clusters of excitatory and inhibitory neurons in mouse and human sSC, we set out to evaluate whether expression levels are correlated between clusters in the two species, a relevant issue when using the mouse to investigate a human disorder. No direct equivalents have been described for the clusters of these two datasets, so we took an indirect route, using additional information from the metadata of the Mouse V1 & ALM - SMART-SEQ (2018) and Human MTG - SMART-SEQ (2018) datasets (both available at https://portal.brain-map.org/atlases-and-data/rnaseq; Extended Data Fig. 2-3*A*). This analysis was only possible for GABAergic neurons, as their clusters (but not the glutamatergic ones) have been correlated between the Whole Cortex & Hippocampus - SMART-SEQ (2019) with 10×-Smart-Seq Taxonomy (2020) and the Mouse V1 & ALM - SMART-SEQ (2018) datasets (Yao et al., 2020). We first determined the composition of the homologous cell types described for these additional mouse and human datasets (Hodge et al., 2019; Extended Data Fig. 2-3*B*), and then determined the correlation between the human middle temporal gyrus (MTG) and Multiple Brain Areas clusters (Extended Data Fig. 2-3*C*). This allowed us to establish an indirect comparison between the clusters with highest *Pcdh19/PCDH19* expression in mouse and human sSC (Table 1). In general, there is a relatively good correlation between the clusters with highest *Pcdh19* expression, particularly for the 3_Lamp Lhx6 cluster, which seems to correspond to chandelier cells in layers V/VI (chandelier type 2 cells; Paul et al., 2017; Tasic et al., 2018), and most (but not all) of the *Vip* clusters and several *Pvalb* clusters, including the chandelier cells of 110_Pvalb Vipr2. Correlation in the *Sst-Chodl* subclass is lower, with mouse long-projecting interneurons expressing higher levels of *Pcdh19* than their human counterparts. Levels of expression in clusters of the *Sst* subclass also tend to show higher variability between the two species.

**Table 1:** Comparison of GABAergic clusters with high *Pcdh19* expression in mouse and human sSC

Mouse Whole Cortex & Hippocampus - SMART-SEQ (2019) with 10×-Smart-Seq Taxonomy (2020)	Mouse V1 & ALM - SMART- SEQ (2018)	Homologous cell type taxonomy (Hodge et al., 2019)	Human MTG - SMART-SEQ (2018)	Human MULTIPLE CORTICAL AREAS - SMART-SEQ (2019)
3_Lamp5 Lhx6 (H)	Lamp5 Lhx6	Lamp5 Lhx6	Inh L2-5 LAMP5 CA1	Inh L1-6 LAMP5 CA13 (H) Inh L5-6 LAMP5 SFTA3 (H)
25_Sncg (M-H) 35_Sncg (H)	Sncg Vip Nptx2 Sncg Gpr50 Sncg Vip Itih5	Vip Sncg	Inh L1-2 VIP TSPAN12	Inh L1 VIP PRSS8 (L)
40-41_Vip (L) 44-47_Vip (L)	Serpinf Aqp5 Vip Vip Pygm C1ql1 Vip Chat Htr1f	Vip 3	Inh L1-2 VIP PCDH20	Inh L1-2 VIP PPAPDC1A (H)
47_Vip (H)	Vip Rspo4 Rxfp1 Chat Vip Rspo1 Itga4	Vip 4	Inh L2-4 VIP CBLN1 Inh L1-3 VIP CCDC184 Inh L1-3 VIP GGH Inh L1-3 VIP CHRM2	Inh L3 VIP CBLN1 (L) Inh L1-3 VIP ACHE (M) Inh L1-3 VIP GGH (H) Inh L1-2 VIP ZNF322P1 (H)
51_Vip (H)	Vip Gpc3 Slc18a3	Vip 2	Inh L2-6 VIP QPCT Inh L3-6 VIP HS3ST3A1	Inh L1-6 VIP RGS16 (H) Inh L2-6 VIP VIP (H) Inh L3-6 VIP KCTD13 (H)
59_Vip (H)	Vip Igfbp6 Car10	Vip 1	Inh L1-4 VIP PENK Inh L1-3 VIP ADAMTSL1 Inh L1-2 SST BAGE2	Inh L1-6 VIP PENK (H) Inh L1-5 VIP KCNJ2 (H) Inh L1 VIP CXCL14 (L) Inh L1 ADARB2 DISP2 (H)
61_Sst Chodl (H)	Sst Chodl	Sst Chodl	Inh L3-6 SST NPY	Inh L6 SST NPY (M)
64_Sst (L) 66_Sst (N.P.) 67_Sst (L) 79_Sst (L) 80-82_Sst (M)	Sst Myh8 Fibin Sst Chrna2 Glra3 Sst Myh8 Etv1 Sst Nr2f2 Necab1 Sst Chrna2 Ptgdr	Sst 1	Inh L3-6 SST HPGD Inh L4-6 SST B3GAT2	Inh L4-6 SST MTHFD2P6 (M)
70_Sst (H) 72_Sst (H) 73_Sst (H) 78_Sst (H)	Sst Tac2 Tacstd2 Sst Rxfp1 Eya1 Sst Rxfp1 Prdm8	Sst 3	Inh L4-6 SST GXYLT2 Inh L5-6 SST NPM1P10	Inh L5-6 SST KLHL14 (L) Inh L5-6 SST ISOC1 (L)
84_Sst (H)	Sst Esm1	Sst 2	Inh L5-6 SST KLHDC8A (only 3 cells)	no equivalent
90_Sst (H) 92_Sst (H) 94_Sst (H) 95_Sst (H)	Sst Calb2 Pdlim5 Sst Tac1 Tacr3 Sst Calb2 Necab1 Sst Tac1 Htr1d	Sst 5	Inh L1-3 SST CALB1	Inh L3-5 SST MAFB (M)
111_Pvalb (H)	Pvalb Akr1c18 Ntf3	Pvalb 1	Inh L5-6 PVALB LGR5 Inh L5-6 SST TH Inh L4-5 PVALB MEPE Inh L5-6 SST MIR548F2	Inh L5-6 PVALB FAM150B (M) Inh L5-6 SST TH (M) Inh L5 PVALB CNTNAP3P2 (M) Inh L5-6 PVALB STON2 (M)
Pvalb Sema3e Kank4 Palb Calb1 Sst	Pvalb 2	Inh L2-4 PVALB WFDC2 Inh L4-6 PVALB SULF1	Inh L2-4 PVALB C8ORF4 (M) Inh L5 PVALB CNTNAP3P2 (M) Inh L1-3 PVALB WFDC2 (H) Inh L3-4 PVALB HOMER3 (L)
112_Pvalb (H)	Pvalb Gpr149 Islr	Pvalb 1	Inh L5-6 PVALB LGR5 Inh L5-6 SST TH Inh L4-5 PVALB MEPE Inh L5-6 SST MIR548F2	Inh L5-6 PVALB FAM150B (M) Inh L5-6 SST TH (M) Inh L5 PVALB CNTNAP3P2 (M) Inh L5-6 PVALB STON2 (M)
113_Pvalb (H) 114_Pvalb (M) 115_Pvalb (H)	Pvalb Tpbg Pvalb Reln Tac1 Pvalb Reln Itm2a	Pvalb 2	Inh L2-4 PVALB WFDC2 Inh L4-6 PVALB SULF1	Inh L2-4 PVALB C8ORF4 (M) Inh L5 PVALB CNTNAP3P2 (M) Inh L1-3 PVALB WFDC2 (H) Inh L3-4 PVALB HOMER3 (L)
116_Pvalb (H)	Sst Tac1 Tacr3 Sst Tac1 Htr1d	Sst 5	Inh L1-3 SST CALB1	Inh L3-5 SST MAFB (M)
Palb Calb1 Sst Pvalb Tpbg	Pvalb 2	Inh L2-4 PVALB WFDC2 Inh L4-6 PVALB SULF1	Inh L2-4 PVALB C8ORF4 (M) Inh L5 PVALB CNTNAP3P2 (M) Inh L1-3 PVALB WFDC2 (H) Inh L3-4 PVALB HOMER3 (L)
119_Pvalb (H)	Pvalb Vipr2	Chandelier	Inh L2-5 PVALB SCUBE3	Inh L1-6 PVALB SCUBE3 (H)

GABAergic clusters with high *Pcdh19* expression in the sSC from either the mouse “Whole Cortex & Hippocampus - SMART-SEQ (2019) with 10×-Smart-Seq taxonomy (2020)” dataset or the human “Multiple Cortical Areas – SMART-SEQ (2019)” dataset are listed in the left and right columns of the table, respectively. The middle columns list the clusters and homologous cell type taxonomy groups that have allowed the indirect correlation between them. H, High expression; M, medium expression; L, low expression; N.P., cluster is not present in the sSC.

### Subtle changes in layer composition in *Pcdh19* mutant animals

Although no major morphologic defects have been described in *Pcdh19* mutant brains (Pederick et al., 2016; Hayashi et al., 2017), a detailed, quantitative study of cortical lamination has not been performed so far. Given that *Pcdh19* is expressed in projection neurons and interneurons, we performed an analysis with markers for both neuronal populations in the somatosensory cortex. We first selected cortical markers for projection neurons of deep and upper layers (CUX1, SATB2, RORB, CTIP2, and TBR1) and performed immunohistochemistry at P10, once radial migration is completed. For each marker, we determined the proportion of positive cells, as well as their distribution within 10 bins covering the whole width of the cortical plate. We analyzed males and females separately, using WT male (WT-M) controls for the KO males and WT female (WT-F) controls for the HET animals (except for CUX1, where this was not possible for technical reasons).

In accordance with previous reports (Pederick et al., 2016), we found no differences in cortical width between genotypes (WT-M average = 1381.47 ± 33.72 μm, KO = 1309.10 ± 32.07 μm, WT-F = 1346.85 ± 39.67 μm, HET = 1348.47 ± 32.46 μm; Fig. 3*A*, Table 2, a). The proportion of positive neurons for all five examined markers was also unaltered (Fig. 3*B*,*C*, Table 2, b–f). CUX1^+^ cells made up approximately one-fifth of all DAPI^+^ cells (WT = 21.24 ± 1.32%, HET = 22.34 ± 1.64%, KO = 24.66 ± 2.05%), and SATB2^+^ cells represented more than one-half of all cells (WT-M = 62.20 ± 4.09%, KO = 58.95 ± 2.45%, WT-F = 63.01 ± 2.78%, HET = 57.96 ± 3.64%). The proportion of RORB^+^ cells seemed lower in KO brains compared with WT-M brains (WT-M = 28.96 ± 0.50%, KO = 18.86 ± 3.74%, WT-F = 27.86 ± 2.15%, HET = 24.37 ± 2.49%), but statistical analysis revealed that this difference was not significant (Mann–Whitney test, *U *=* *3, *p *=* *0.2). CTIP2^+^ cells were also equally abundant among the four groups (WT-M = 19.97 ± 3.94%, KO = 13.58 ± 1.15%, WT-F = 18.81 ± 3.16%, HET = 15.89 ± 2.46%), and TBR1^+^ cells added up to approximately one-third of all cells (WT-M = 32.40 ± 2.26%, KO = 38.43 ± 1.80%, WT-F = 35.21 ± 2.40%, HET = 33.85 ± 2.64%).

**Table 2:** Statistical analysis of cortical width and marker composition at P10

Data	Comparison (*n*)	Data structure (normality?)	Equal variance?	Test	Results
Cortical width (a)	WT-M (7) vs KO-M (5)	Yes	Yes	Unpaired *t* test	*t*_(2,10)_ = 1.495 *p *=* *0.1658
	WT-F (7) vs HET-F (9)	No	Yes	Mann–Whitney	*U *=* *31 *p *>* *0.9999
	WT-M (7) vs WT-F (7)	Yes	Yes	Unpaired *t* test	*t*_(2,12)_ = 0.6648 *p *=* *0.5187
% CUX1 over DAPI (b)	WT (4) vs KO (4) vs HET (4)	Yes	Yes	one-way ANOVA	*F*_(2,9)_ = 1.065 *p *=* *0.3846
% RORB over DAPI (c)	WT-M (4) vs KO-M (4)	No	Yes	Mann–Whitney	*U *=* *3 *p *=* *0.2
	WT-F (4) vs HET-F (4)	Yes	Yes	Unpaired *t* test	*t*_(2,6)_ = 1.060 *p *=* *0.3301
	WT-M (4) vs WT-F (4)	No	Yes	Mann–Whitney	*U *=* *7 *p *=* *0.8857
% SATB2 over DAPI (d)	WT-M (4) vs KO-M (4)	Yes	Yes	Unpaired *t* test	*t*_(2,6)_ = 0.6827 *p *=* *0.5203
	WT-F (4) vs HET-F (4)	Yes	Yes	Unpaired *t* test	*t*_(2,6)_ = 1.105 *p *=* *0.3113
	WT-M (4) vs WT-F (4)	Yes	Yes	Unpaired *t* test	*t*_(2,6)_ = 0.1644 *p *=* *0.8749
% CTIP2 over DAPI (e)	WT-M (4) vs KO-M (4)	Yes	Yes	Unpaired *t* test	*t*_(2,6)_ = 1.557 *p *=* *0.1704
	WT-F (4) vs HET-F (4)	Yes	Yes	Unpaired *t* test	*t*_(2,6)_ = 0.7295 *p *=* *0.4932
	WT-M (4) vs WT-F (4)	Yes	Yes	Unpaired *t* test	*t*_(2,6)_ = 0.2306 *p *=* *0.8253
% TBR1 over DAPI (f)	WT-M (4) vs KO-M (4)	No	Yes	Mann–Whitney	*U *=* *1 *p *=* *0.0571
	WT-F (4) vs HET-F (4)	Yes	Yes	Unpaired *t* test	*t*_(2,6)_ = 0.3816 *p *=* *0.7159
	WT-M (4) vs WT-F (4)	Yes	Yes	Unpaired *t* test	*t*_(2,6)_ = 0.8509 *p *=* *0.4275

The table lists the data analyzed and the groups that have been compared, including the number of independent samples. Normality of the data and equality of variance for the groups compared are indicated, as well as the statistical test performed and the obtained results. The details of the tests performed for the layer distribution of individual markers have not been included, for simplicity.

The distribution of SATB2^+^ neurons between the 10 bins was unchanged for males and females (Fig. 3*G–J*). However, we detected some deviations in the distribution of CUX1^+^, CTIP2^+^, RORB^+^, and TBR1^+^ neurons (Fig. 3*D–L*). Regarding CUX1, the difference was apparent in bin 5 (Fig. 3*E*). *Pcdh19*-HET animals showed a significant 2.4-fold reduction in the percentage of CUX1^+^ neurons in this bin compared with wild types (WT = 2.08 ± 0.18%, HET = 0.86 ± 0.27%, KO = 1.14 ± 0.32%; one-way ANOVA, *F*_(2,9)_ = 5.81, *p *=* *0.0239; Tukey’s test: *q*_(1,9)_ = 4.60, *p *=* *0.0245 HET vs WT). For CTIP2, we found differences in bins 3 (1.7-fold increase) and 7 (1.6-fold reduction) in KO males, suggesting a redistribution of CTIP2^+^ neurons to higher positions in layer V (bin 3: WT-M = 2.76 ± 0.37%, KO-M = 4.17 ± 0.34%; independent *t* test, *t*_(2,6)_ = 2.787, *p *=* *0.0317; bin 7: WT-M = 16.74 ± 1.67%, KO-M = 10.68 ± 0.34%; independent *t* test with Welch correction for unequal variance, *t *=* *3.556, *p *=* *0.0333). HET females showed double the percentage of cells in bin 1 than their WT siblings (WT-F = 2.20 ± 0.29%, HET-F = 4.42 ± 0.29%; independent *t* test, *t*_(2,6)_ = 5.391, *p *=* *0.0017; Fig. 3*D*,*F*). Differences in RORB^+^ distribution were only present in males, specifically in bin 2, with a 3.4-fold reduction (WT-M = 11.38 ± 2.00%, KO-M = 3.36 ± 2.37%; independent *t* test, *t*_(2,6)_ = 2.585, *p *=* *0.0415; Fig. 3*G*,*H*). However, the graphs for KO and HET animals suggest that the distribution of RORB^+^ cells tended to be more condensed in those animals. Finally, KO males showed a 2.4-fold increase in the percentage of TBR1^+^ cells in bin 1 compared with their WT counterparts (WT-M = 1.77 ± 0.33%, KO-M = 4.50 ± 0.33%; independent *t* test, *t*_(2,6)_ = 5.818, *p *=* *0.0011), and HET females had a 1.4-fold reduction in the percentage of TBR1^+^ cells in bin 3 (WT-F = 15.98 ± 0.58%, HET-F = 11.10 ± 0.92%; independent *t* test, *t*_(2,6)_ = 4.473, *p *= 0.0042) and a 1.6-fold increase in bin 5 (WT-F = 4.62 ± 0.79%, HET-F = 7.46 ± 0.35%; independent *t* test, *t*_(2,6)_ = 3.268, *p *=* *0.0171; Fig. 3*K*,*L*). A comparison between WT males and females did not reveal any differences in the distribution of the four markers analyzed for excitatory neurons (data not shown).

**Figure 4.**
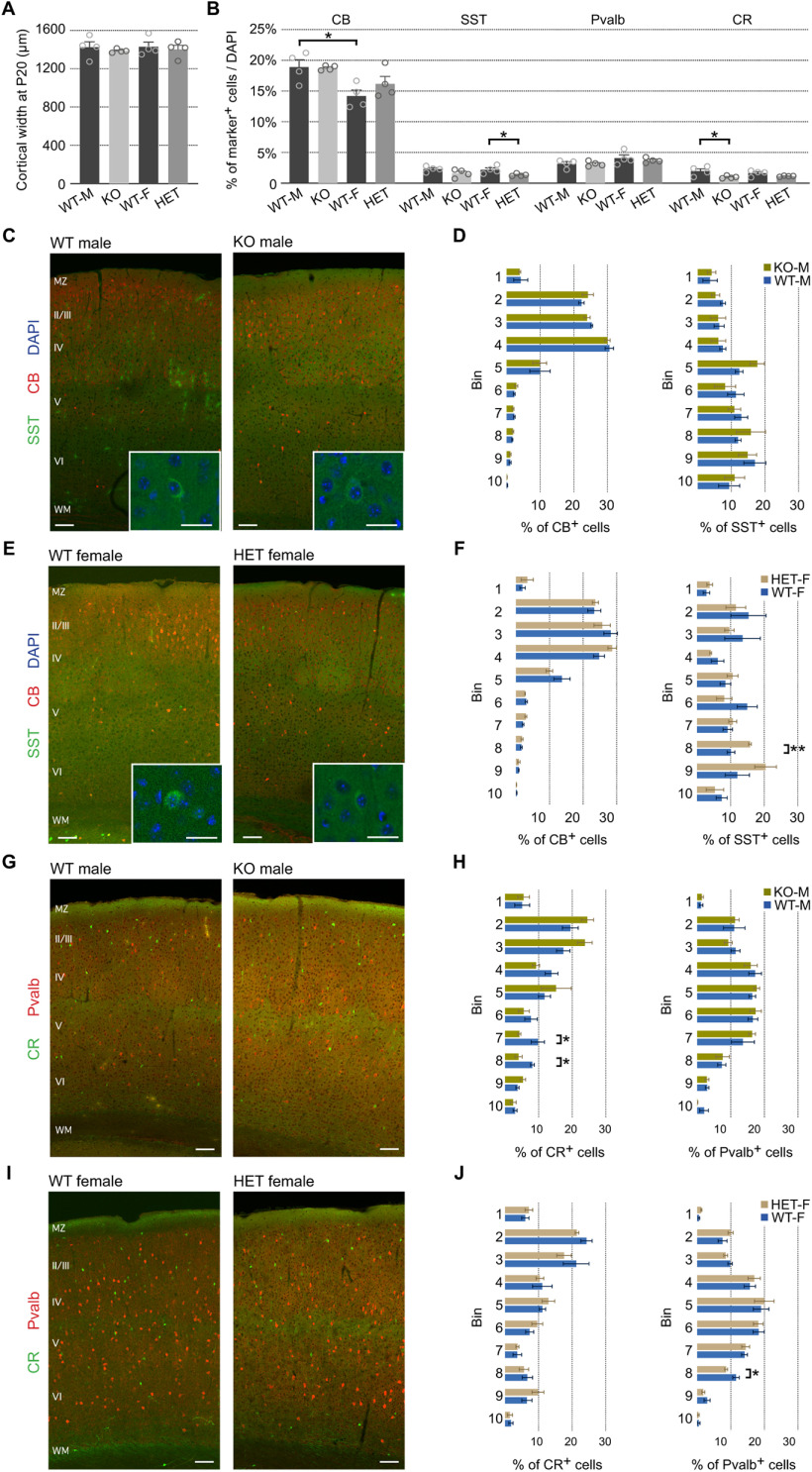
Subtle changes in the distribution of inhibitory neurons in the cortex of *Pcdh19* mutant animals. ***A***, Quantification of cortical width at P20 in *Pcdh19* WT and mutant animals, separated by sex. ***B***, Relative percentages of the different cortical markers examined with respect to total DAPI^+^ cells in the somatosensory cortex. Analysis performed separately for males and females. ***C***, ***E***, Representative confocal micrographs of immunohistochemistry with anti-calbindin (CB; red) and anti-SST (green) antibodies on WT and KO male tissue (***C***), and WT and HET female tissue (***E***). Insets, High-magnification image of SST^+^ cells. Nuclei were counterstained with DAPI (blue). ***D***, ***F***, Quantification of the percentage of CB^+^ (left) and SST^+^ (right) cells in each of 10 equal bins spanning the cortical wall for males (***D***) and females (***F***). ***G***, ***I***, Representative confocal micrographs of immunohistochemistry with anti-parvalbumin (Pvalb; red) and anti-CR (green) antibodies on WT and KO male tissue (***G***), and WT and HET female tissue (***I***). ***H***, ***J***, Distribution of CR^+^ (left) and Pvalb^+^ (right) cells in each of 10 equal bins spanning the cortical wall, shown as a percentage; male data are shown in ***H***, and female data are shown in ***J***. All results are indicated as the mean ± SEM. A minimum of three images per brain, obtained from four animals originating from three different litters were analyzed for each condition. *p < 0.05, **p < 0.01. Scale bars: 200 μm; insets, 50 μm.

To complete our analysis on cortical composition and lamination, we stained the sSC with four different interneuronal markers (SST, PVALB, CB, and CR) in P20 brains. As before, cortical thickness showed no difference between genotypes of matched sex (WT-M average = 1424.49 ± 57.19 μm, KO = 1387.02 ± 9.88 μm, WT-F = 1429.61 ± 48.84 μm, HET = 1402.97 ± 42.92 μm; Fig. 4*A*, Table 3, a). However, in this case, some differences were apparent in the overall proportion of three types of interneurons, which may be due in part to the smaller number of cells that test positive for these markers (Fig. 4*B*, Table 3, b–e). The most abundant type was CB^+^ cells (WT-M = 18.91 ± 1.20%, KO = 18.77 ± 0.20%, WT-F = 14.19 ± 0.98%, HET = 16.20 ± 1.21%), which, despite no changes between genotypes within males or females, displayed a significantly lower proportion in WT females than in WT males (unpaired *t* test, *t*_(2,6)_ = 3.054, *p *=* *0.0224). PVALB^+^, SST^+^, and CR^+^ accounted for <5% of DAPI^+^ cells each (Fig. 4*B*). The proportion of PVALB^+^ interneurons was very similar across the four groups (WT-M = 3.16 ± 0.33%, KO = 3.15 ± 0.21%, WT-F = 4.06 ± 0.55%, HET = 3.70 ± 0.20%), but HET females showed a slight decrease in SST^+^ cells (WT-M = 2.31 ± 0.23%, KO = 1.61 ± 0.33%, WT-F = 2.19 ± 0.31%, HET = 1.34 ± 0.11%; unpaired *t* test, WT-F vs HET: *t*_(2,6)_ = 2.578, *p *=* *0.0419) and KO males a similarly small decrease in CR^+^ interneurons (WT-M = 1.98 ± 0.39%, KO = 0.98 ± 0.10%, WT-F = 1.63 ± 0.24%, HET = 1.14 ± 0.04%; unpaired *t* test, WT-M vs KO, *t*_(2,6)_ = 2.509, *p *=* *0.0459).

**Table 3:** Statistical analysis of cortical width and marker composition at P20

Data	Comparison (*n*)	Data structure (normality?)	Equal variance?	Test	Results
Cortical width (a)	WT-M (4) vs KO-M (4)	Yes	No	Welch’s *t* test	*t*_(2,3.179)_ = 0.6456 *p *=* *0.1658
	WT-F (4) vs HET-F (4)	Yes	Yes	Unpaired *t* test	*t*_(2,6)_ = 0.4098 *p *=* *0.6962
	WT-M (4) vs WT-F (4)	Yes	Yes	Unpaired *t* test	*t*_(2,6)_ = 0.06806 *p *=* *0.9480
% CB over DAPI (b)	WT-M (4) vs KO-M (4)	Yes	No	Welch's *t* test	*t*_(2,3.168)_ = 0.1169 *p *=* *0.9140
	WT-F (4) vs HET-F (4)	Yes	Yes	Unpaired *t* test	*t*_(2,6)_ = 1.291 *p *=* *0.2443
	WT-M (4) vs WT-F (4)	Yes	Yes	Unpaired *t* test	*t*_(2,6)_ = 3.054 *p *=* *0.0224
% SST over DAPI (c)	WT-M (4) vs KO-M (4)	Yes	Yes	Unpaired *t* test	*t*_(2,6)_ = 1.733 *p *=* *0.1339
	WT-F (4) vs HET-F (4)	Yes	Yes	Unpaired *t* test	*t*_(2,6)_ = 2.578 *p *=* *0.0419
	WT-M (4) vs WT-F (4)	Yes	Yes	Unpaired *t* test	*t*_(2,6)_ = 0.3061 *p *=* *0.7698
% PVALB over DAPI (d)	WT-M (4) vs KO-M (4)	Yes	Yes	Unpaired *t* test	*t*_(2,6)_ = 0.01984 *p *=* *0.9848
	WT-F (4) vs HET-F (4)	Yes	Yes	Unpaired *t* test	*t*_(2,6)_ = 0.6266 *p *=* *0.5540
	WT-M (4) vs WT-F (4)	Yes	Yes	Unpaired *t* test	*t*_(2,6)_ = 1.421 *p *=* *0.2051
% CR over DAPI (e)	WT-M (4) vs KO-M (4)	Yes	Yes	Unpaired *t* test	*t*_(2,6)_ = 0.0459 *p *=* *2.509
	WT-F (4) vs HET-F (4)	Yes	No	Welch's *t* test	*t*_(2,3.172)_ = 2.026 *p *=* *0.1308
	WT-M (4) vs WT-F (4)	Yes	Yes	Unpaired *t* test	*t*_(2,6)_ = 0.7616 *p *=* *0.4752

The table includes the data analyzed and the comparisons made, listing the number of independent samples. Normality of the data and equality of variance for the groups compared are included, as well as the statistical test performed and the obtained results. The details of the tests performed for the layer distribution of individual markers have not been included, for simplicity.

Regarding cellular distribution in the sSC, no differences were apparent for CB^+^ cells in KO males or HET females (Fig. 4*C–F*). However, we detected changes in the distribution of SST^+^ (HET females), CR^+^ (KO males), and PVALB^+^ (HET females) interneurons (Fig. 4*C*–*J*). HET brains displayed a 1.6-fold increase in the percentage of SST^+^ cells in bin 8 when compared with gender matched WT brains (WT-F = 10.13 ± 1.15%, HET-F = 15.79 ± 0.4%; independent *t* test, *t*_(2,6)_ = 4.647, *p *=* *0.0035; Fig. 4*E*,*F*). Although not significant because of higher variability, bin 9 also reflects an increase in SST^+^ interneurons in HET brains, whereas bins 2 and 3 seem to have reduced numbers, suggesting a potential redistribution of SST^+^ cells toward deeper layers in HET females. Changes in CR^+^ cell distribution were found in bin 8 of KO brains, which displayed an approximately twofold reduction over WT male brains (bin 8: WT-M = 8.16 ± 0.57%, KO-M = 4.06 ± 1.05%; Mann–Whitney test, *p *=* *0.0286; Fig. 4*G*,*H*). This change, combined with another decrease in bin 7 and concomitant increases in bins 2 and 3 that did not reach statistical significance, might indicate a tendency of CR^+^ interneurons to occupy higher positions within the cortex in KO animals. As for PVALB^+^ cells, HET brains showed a reduced percentage in bin 8 (WT-F = 11.54 ± 0.96%, HET-F = 8.61 ± 0.44%; independent *t* test, *t*_(2,6)_ = 2.777, *p *=* *0.0321; Fig. 4*I*,*J*). In this case, some differences were found in the distribution of CB^+^ (bin 4), CR^+^ (bin 8), and PVALB^+^ (bin 7) interneurons between WT males and females (data not shown, but see Discussion).

In summary, despite relative neuronal proportions and distributions being mostly normal in the sSC of *Pcdh19* mutant animals, subtle but significant differences in distribution are apparent for many of the analyzed neuronal markers.

### No obvious defects in axonal tracts in *Pcdh19* mutant animals

**Figure 5.**
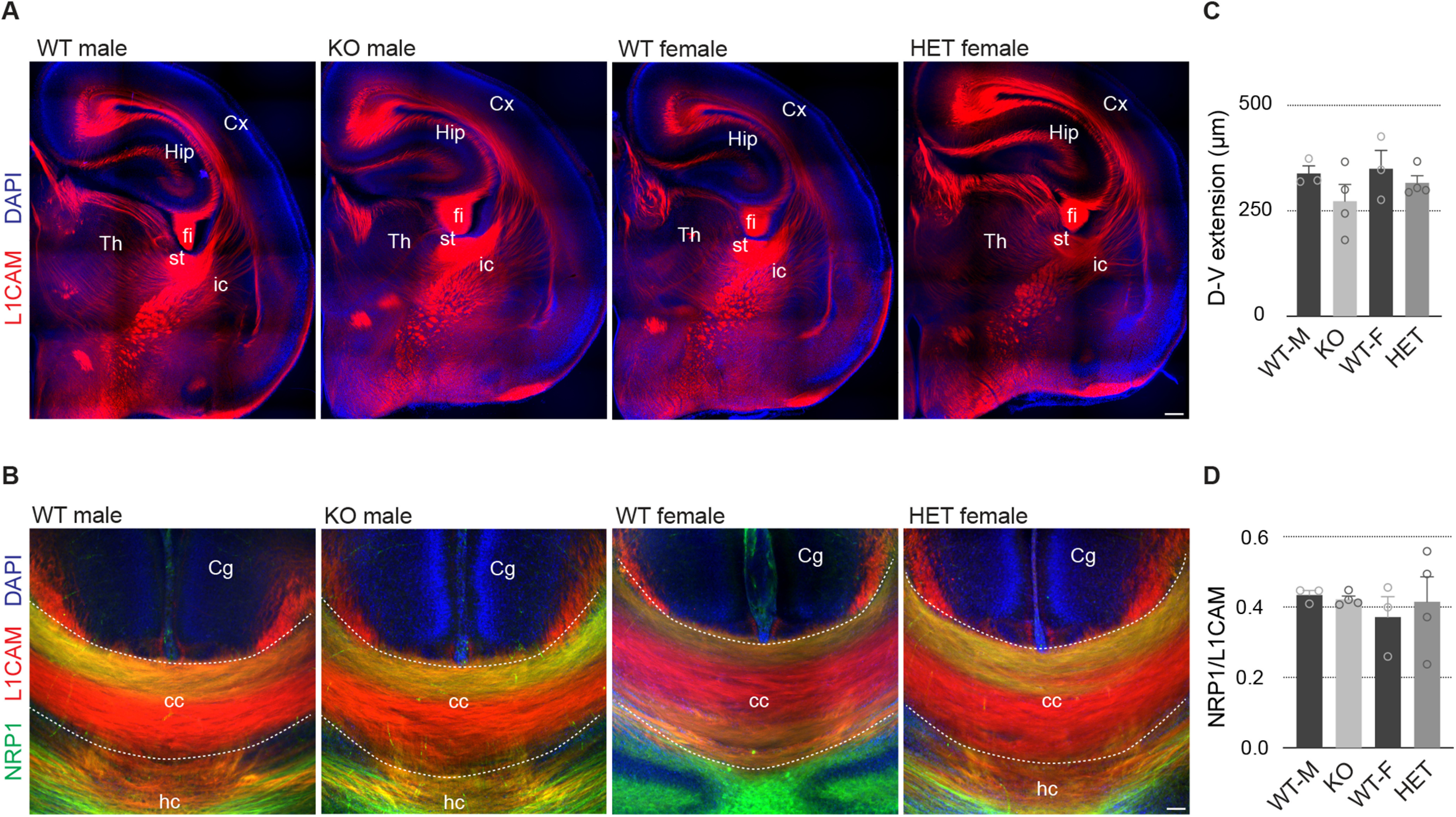
No major anomalies in the main axonal tracts in *Pcdh19* mouse mutants. ***A***, Confocal micrographs of P0–P1 mouse hemispheres stained with anti-L1CAM (red). Nuclei were counterstained with DAPI (blue). ***B***, Confocal micrographs of the corpus callosum of P0–P1 mice stained with anti-L1CAM (red) and anti-Neuropilin-1 (green), and counterstained with DAPI (blue). ***C***, Quantification of the dorsoventral extension of the corpus callosum in WT and mutant animals, separated by sex. ***D***, Quantification of the dorsal restriction of Neuropilin-1^+^ axons in WT and mutant animals, separated by sex. All results are indicated as the mean ± SEM. Two images per brain, obtained from four animals originating from three different litters, were analyzed for each condition. Cx, Cortex; Hip, hippocampus; Th, thalamus, fi, fimbria; st, striatum; ic, internal capsule; Cg, cingulate cortex; cc, corpus callosum; hc, hippocampal commissure. Scale bars: ***A***, 200 μm; ***B***, 50 μm.

Our results indicate that *Pcdh19* is expressed in cortical projection neurons that project through the corpus callosum (layer II–III and some layer V neurons), as well as in neurons projecting outside the cortex, mainly through the pyramidal tract (layer V PT neurons). Although several members of the cadherin superfamily, including δ-protocadherins 7, 10, 17, and 18, have been shown to play a role in axonal outgrowth (Uemura et al., 2007; Piper et al., 2008; Hayashi et al., 2014), fasciculation (Williams et al., 2011; Hayashi et al., 2014), and arborization (Biswas et al., 2014), it is not known whether mutations in *Pcdh19* have an impact on any of these processes. We therefore conducted a general characterization of axonal tracts in Taconic Biosciences *Pcdh19* male and female WT, male KO, and female HET animals by immunostaining against the cell adhesion molecule L1CAM (Fig. 5*A*). No differences were apparent for males or females between genotypes in the major axonal tracts, including the internal capsule, stria terminalis, fimbria, or corpus callosum. Next, we analyzed the corpus callosum in more detail by labeling dorsally located axons with Neuropilin-1, which allows the analysis of topographical organization at the midline. Again, the dorsoventral extension of the corpus callosum and the dorsal restriction of Neuropilin-1-expressing axons was similar between genotypes for both male and female animals (Fig. 5*B–D*, Table 4, a and b). Thus, our results revealed no major abnormalities in the main axonal tracts, although they do not preclude the existence of more subtle defects that would require a more detailed analysis to be revealed.

**Table 4:** Statistical analysis of dorsoventral extension and NRP1/L1CAM ratio in the corpus callosum of wild-type and *Pcdh19* mutant pups

Data	Comparison (*n*)	Data structure (normality?)	Equal variance?	Test	Results
D-V extension (a)	WT-M (3) vs KO-M (4)	Yes	Yes	Unpaired *t* test	*t*_(2,5)_ = 1.338 *p *=* *0.2385
	WT-F (3) vs HET-F (4)	No	Yes	Mann–Whitney	*U *=* *5 *p *=* *0.8571
	WT-M (3) vs WT-F (3)	Yes	Yes	Unpaired *t* test	*t*_(2,4)_ = 0.2420 *p *=* *0.8206
NRP1/L1CAM ratio (b)	WT-M (3) vs KO-M (4)	No	Yes	Mann–Whitney	*U *=* *5 *p *=* *0.8571
	WT-F (3) vs HET-F (4)	Yes	Yes	Unpaired *t* test	*t*_(2,5)_ = 0.4525 *p *=* *0.6699
	WT-M (3) vs WT-F (3)	No	Yes	Mann–Whitney	*U *=* *3 *p *=* *0.7000

The table lists the data analyzed and the groups that have been compared, including the number of independent samples. Normality of the data and equality of variance for the groups compared are indicated, as well as the statistical test performed and the obtained results.

### Altered behavior in *Pcdh19* mutant animals and their littermates

**Figure 6.**
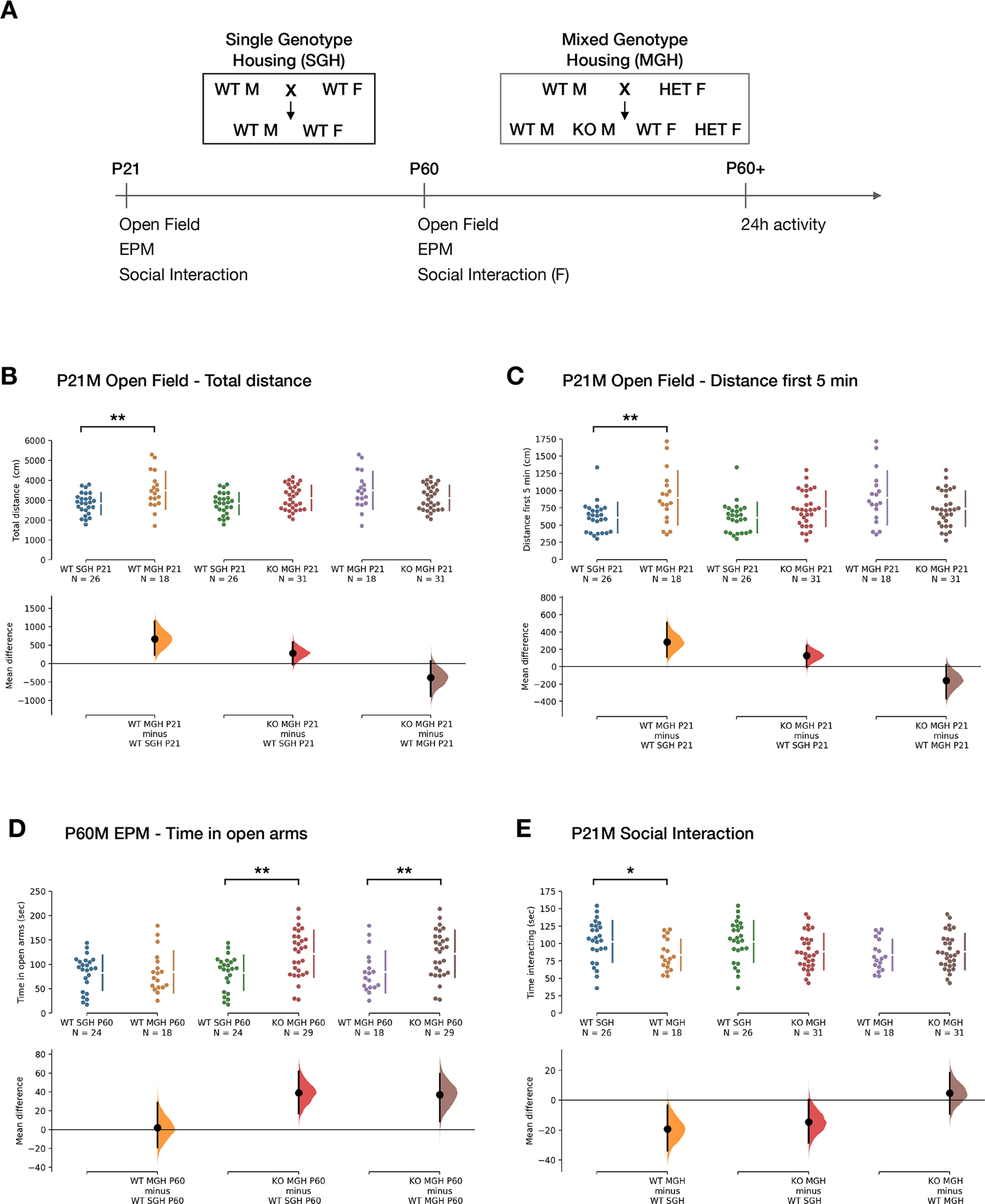
Behavioral alterations in *Pcdh19* KO males and their WT littermates. ***A***, Schematic of the behavioral experiments conducted. ***B***, Total distance traveled by males during the 20 min of the open field test at P21. ***C***, Distance traveled in the open field test by P21 males in the first 5 min interval of the open field test. Open field test results correspond to the second day of testing in ***A*** and ***B***. ***D***, Total time spent by males in the open arms of the elevated plus maze during the 5 min test at P60. ***E***, Time spent by P21 males interacting with a nonfamiliar female in estrus. The total duration of the test was 5 min. ***B–D***, The top axis shows the raw data points for each group. To their right, the gap in the line indicates the mean, and the lines extending vertically represent the SD. The group and group sizes are indicated at the bottom. Note that each group appears twice in every graph, but with two different colors. The mean difference for each comparison is plotted in the lower axis as a bootstrap sampling distribution. The black dot represents the mean, and the vertical bar its 95% confidence interval. At the top of each graph the significance scores of the one-way ANOVA or Kruskal–Wallis test and their *post hoc* test are indicated. *p < 0.05; **p < 0.01. Test results with male animals that did not reach significance are presented in Extended Data Figure 6-1.

While there are no major lamination defects in the cortex and in the main axonal tracts of the brain of *Pcdh19* mutant animals, the changes in the distribution of specific neuronal subtypes revealed by our quantitative analysis could lead to local connectivity defects that could become apparent at the behavioral level. Indeed, synaptic defects have recently been described between *Pcdh19* WT and KO neurons (Hoshina et al., 2021; Mincheva-Tasheva et al., 2021). Thus, we also conducted a series of tests to determine whether these animals present any behavioral alterations. The paradigms included the open field test to evaluate general locomotor activity, anxiety, and exploratory behavior; elevated plus maze (EPM) test to measure anxiety; and a social interaction test. We assessed animals at preweaning age and as adults, to account for any developmental effects. In addition to the WT littermates that *Pcdh19* mutant animals were housed with, we included a further control of SGH WT animals (WT^SGH^; Fig. 6*A*). Indeed, we note that a previous study on the X-linked ASD-related gene *Nlgn3*, also a membrane protein expressed in the developing cerebral cortex, revealed that housing conditions impact the behavior of wild-type animals when housed together with mutant animals (Kalbassi et al., 2017). The parents of the animals used to analyze behavior in the single-genotype housed WT condition originated from our *Pcdh19* colony, and behavior was analyzed separately for male and female mice. For the behavioral analysis, we have added estimation statistics with CIs to the more common statistical inference analysis (one-way ANOVA or Kruskal–Wallis test among the three groups) to improve the interpretation of results. Because estimation statistics compare the means of only two groups, we provide the average mean difference (M_diff_) and unbiased Cohen’s *d* of the particular comparison with their corresponding 95% confidence intervals, followed by the results of the overall comparison with ANOVA or Kruskal–Wallis test and the relevant *post hoc* analysis. When the means of the three groups were not deemed different by any of the two methods, we only present the common statistical inference analysis for brevity.

10.1523/ENEURO.0510-20.2021.f6-1 Figure 6-1 ***A***, Total distance travelled in the open field test by P60 males. ***B***, Distance travelled in the open field test by P21 males, split into 5 min intervals. The data for the first 5 min are shown in Figure 6. ***C***, Distance travelled in the open field test by P60 males, split into 5 min intervals. ***D***, Time spent by males in the center of the arena during the 20 min open field test at P21 and P60. ***E***, Time spent in the open arms of the elevated plus maze by P21 males. ***F***, Number of beam breaks during the 24 h activity test for the males of the different conditions. Light, light phase; dark, dark phase. ***G***, Number of beam breaks by hour in the 24 h activity test for the males of the different conditions. The time of the day is shown on the *x*-axis, and the gray square indicates the hours of the dark period. The numbers of tested animals were as follows: at P21: WT^SGH^, 26; WT^MGH^, 18; KO, 31; at P60: WT^SGH^, 24; WT^MGH^, 18; KO, 29. For the 24 h activity test, the numbers were as follows: WT^SGH^, 17; WT^MGH^, 10; KO, 10. Results are indicated as the mean ± SEM. *p < 0.05. Download Figure 6-1, TIF file.

Differences in male behavior were evident at P21 (Fig. 6*B–E*, Table 5). MGH WT (WT^MGH^) males traveled on average 23% more distance during the 20 min open field paradigm than WT^SGH^ males. The unpaired M_diff_ was 667.54 cm (95% CI, 233.04, 1150.34; Fig. 6*B*) and the unbiased Cohen’s *d* for this comparison was 0.89 (95% CI, 0.29, 1.59), indicating a strong effect of housing (one-way ANOVA, *F*_(2,72)_ = 5.02, *p *=* *0.0091; *post hoc* Tukey’s test, WT^MGH^ vs WT^SGH^: *q*_(1,72)_ = 4.48, *p *=* *0.0063). In this experiment, KO animals also traveled a higher distance than WT^SGH^ (M_diff_ = 281.06 cm, 95% CI, −25.36, 576.08), but an effect of genotype cannot be confirmed with these data. An analysis by 5 min slots showed that the increased distance traveled by WT^MGH^ males compared with WT^SGH^ males was mainly because of a 47% increase in activity during the first 5 min [M_diff_ = 285.95 cm; 95% CI, 112.15, 510.92; unbiased Cohen’s *d *=* *0.94 (95% CI, 0.34, 1.65); Kruskal–Wallis test: *H*_(2)_ = 9.35, *p *=* *0.0093; *post hoc* Dunn’s test, WT^MGH^ vs WT^SGH^: *z *=* *3.01, *p *=* *0.0079; Fig. 6*C*]. Although KO males showed a 21% increase in activity during this period when compared with WT^SGH^ males [M_diff_ = 127.75 cm; 95% CI, −4.02, 243.94; unbiased Cohen’s *d *=* *0.53 (95% CI, 0, 1.09)], this difference again does not seem to reflect a real change in behavior, suggesting that increased activity might be an effect of housing in males, rather than of genotype (Kruskal–Wallis test: *H*_(2)_ = 9.35, *p *=* *0.0093; *post hoc* Dunn’s test, KO vs WT^SGH^: *z *=* *3.01, *p *=* *0.1711; Fig. 6*C*). The increased activity of WT^MGH^ males over WT^SGH^ males disappeared after the first 5 min and also when animals were tested again at ≥P60 [total distance M_diff_ = 351.78 cm (95% CI, −197.54, 934.76); unbiased Cohen’s *d *=* *0.36 (95% CI, −0.26, 1.02); one-way ANOVA: *F*_(2,68)_ = 1.13, *p *=* *0.329; first 5 min: M_diff_ = 84.50 cm (95% CI, −96.61, 256.31); unbiased Cohen’s *d *=* *0.27 (95% CI, −0.35, 0.92); one-way ANOVA: *F*_(2,68)_ = 1.31, *p *=* *0.2759; Extended Data Fig. 6-1*A–C*]. In accordance with these results, spontaneous activity (number of beam breaks) over a 24 h period in adult male mice did not differ significantly between conditions (Extended Data Fig. 6-1*F*,*G*), when analyzed in total (one-way ANOVA: *F*_(2,34)_ = 0.48, *p *=* *0.621), in the light (one-way ANOVA: *F*_(2,34)_ = 3.03, *p *=* *0.0615), or in the dark period (one-way ANOVA: *F*_(2,34)_ = 0.31, *p *=* *0.733). Isolated differences at individual time points (7:00 P.M.: Kruskal–Wallis test, *H*_(2)_ = 16.08, *p *=* *0.0003; *post hoc* Dunn’s test, KO vs WT^MGH^: *z *=* *4.01, *p *=* *0.0002; 8:00 P.M.: one-way ANOVA, *F*_(2,34)_ = 5.18, *p *=* *0.0109; *post hoc* Tukey’s test, HET vs KO vs WT^SGH^: *q*_(1,34)_ = 4.42, *p *=* *0.0099; 10:00 A.M.: Kruskal–Wallis test: *H*_(2)_ = 10.78, *p *=* *0.0046; *post hoc* Dunn’s test, KO vs WT^MGH^: *z *=* *3.11, *p *=* *0.0056; KO vs WT^SGH^: *z *=* *2.62, *p *=* *0.0267; 8:00 A.M.: Kruskal–Wallis test, *H*(2) = 7.17, *p *=* *0.0277; *post hoc* Dunn’s test, WT^MGH^ vs KO: *z *=* *2.51, *p *=* *0.0361; Extended Data Fig. 6-1*G*) do not seem to point to an overall activity defect and might be because of a smaller number of animals being tested.

**Table 5:** Statistical analysis of the behavioral experiments in P21 and adult males

Behavioral test	Sex	Age	Normal data?	Equal variance?	Test	Results
Open field test: total distance day 2	M	P21	Yes	Yes	One-way ANOVA	*F*_(2,72)_ = 5.017; *p *=* *0.0091 *Post hoc* Tukey’s test: WT^SGH^ vs WT^MGH^; *p *=* *0.0063 WT^SGH^ vs KO; *p *=* *0.2796 WT^MGH^ vs KO; *p *=* *0.1468
Open field test: total distance day 2	M	P60	Yes	Yes	One-way ANOVA	*F*_(2,68)_ = 1.13; *p *=* *0.329
Open field test: intervals day 2: 0–5 min	M	P21	No	Yes	Kruskal–Wallis	*H*_(2)_ = 9.354; *p *=* *0.0093 *Post hoc* Dunn’s test: WT^SGH^ vs WT^MGH^; *p *=* *0.0079 WT^SGH^ vs KO; *p *=* *0.1711 WT^MGH^ vs KO; *p *=* *0.4797
Open field test: intervals day 2: 5–10 min	M	P21	Yes	Yes	One-way ANOVA	*F*_(2,72)_ = 0.719; *p *=* *0.491
Open field test: intervals day 2: 10–15 min	M	P21	Yes	Yes	One-way ANOVA	*F*_(2,72)_ = 0.976; *p *=* *0.382
Open field test: intervals day 2: 15–20 min	M	P21	Yes	Yes	One-way ANOVA	*F*_(2,72)_ = 2.184; *p *=* *0.12
Open field test: intervals day 2: 0–5 min	M	P60	Yes	Yes	One-way ANOVA	*F*_(2,68)_ = 1.312; *p *=* *0.276
Open field test: intervals day 2: 5–10 min	M	P60	Yes	Yes	One-way ANOVA	*F*_(2,68)_ = 1.292; *p *=* *0.2813
Open field test: intervals day 2: 10–15 min	M	P60	Yes	Yes	One-way ANOVA	*F*_(2,68)_ = 0.13; *p *=* *0.879
Open field test: intervals day 2: 15–20 min	M	P60	No	Yes	Kruskal Wallis	*H*_(2)_ = 1.56; *p *=* *0.4584
Open field test: time in left day 2	M	P21	No	Yes	Kruskal Wallis	*H*_(2)_ = 2.7579; *p *=* *0.2518
Open field test: time in left day 2	M	P60	No	Yes	Kruskal Wallis	*H*_(2)_ = 3.2761; *p *=* *0.1671
24 h activity: total	M	>P60	Yes	Yes	One-way ANOVA	*F*_(2,34)_ = 0.4831; *p *=* *0.6210
24 h activity: light period	M	>P60	Yes	Yes	One-way ANOVA	*F*_(2,34)_ = 3.031; *p *=* *0.0615
24 h activity: dark period	M	>P60	Yes	Yes	One-way ANOVA	*F*_(2,34)_ = 0.3135; *p *=* *0.7330
Elevated plus maze	M	P21	Yes	Yes	One-way ANOVA	*F*_(2,72)_ = 1.994; *p *=* *0.144
Elevated plus maze	M	P60	Yes	Yes	One-way ANOVA	*F*_(2,68)_ = 6.879; *p *=* *0.0019 *Post hoc* Tukey’s test: WT^SGH^ vs WT^MGH^; *p *=* *0.9893 WT^SGH^ vs KO; *p *=* *0.0042 WT^MGH^ vs KO; *p *=* *0.0138
Social interaction	M	P21	Yes	Yes	One-way ANOVA	*F*_(2,72)_ = 2.911; *p *=* *0.039 *Post hoc* Dunnett’s test (all vs WT SGH): WT^SGH^ vs WT^MGH^; *p *=* *0.0382 WT^SGH^ vs KO; *p *=* *0.0771

The table includes the behavioral test analyzed, sex and age of the animals, data and variance distribution, statistical test used, and results obtained. M, Male.

To investigate whether the increased distance traveled by preweaned mixed-genotype housed WT animals in the first 5 min of the open field test could be because of increased anxiety, we analyzed the time spent in the center of the arena. No differences were found among the three conditions, either at P21 (Kruskal–Wallis test: *H*_(2)_ = 2.76, *p *=* *0.2518) or at P60 (Kruskal–Wallis test: *H*_(2)_ = 3.58, *p *=* *0.1671; Extended Data Fig. 6-1*D*). The results of the elevated plus maze confirmed the lack of differences at P21 (Kruskal–Wallis test: *H*_(2)_ = 4.57, *p *=* *0.1016; Extended Data Fig. 6-1*E*). However, this was not the case for adult animals, as adult KO males spent >40% more time in the open arms than their WT^MGH^ littermates and WT^SGH^ controls, pointing to an effect of genotype in reducing anxiety [KO vs WT^MGH^: M_diff_ = 37.21 s; 95% CI, 8.57, 59.45; unbiased Cohen’s *d *=* *0.80 (95% CI, 0.21, 1.46); KO vs WT^SGH^: M_diff_ = 39.07 s (95% CI, 16.96, 61.53); unbiased Cohen’s *d *=* *0.90 (95% CI, 0.35, 1.52); one-way ANOVA: *F*_(2,68)_ = 6.88; *p *=* *0.0019; Tukey’s test, KO vs WT^MGH^: *q*_(1,68)_ = 4.10, *p *=* *0.0138; KO vs WT^SGH^: *q*_(1,68)_ = 4.68, *p *=* *0.0042; Fig. 6*D*].

Interestingly, we also detected a subtle difference in social behavior at P21. In this case, WT^MGH^ males spent 19% less time interacting with an unfamiliar female in estrus than single-genotype housed WT males [M_diff_ = −19.26 s; 95% CI, −33.73, −3.32; unbiased Cohen’s *d* = −0.70 (95% CI, −1.38, −0.10); one-way ANOVA: *F*_(2,72)_ = 3.39; *p *=* *0.039; Dunnett’s test, WT^MGH^ vs WT^SGH^: *q*_(1,72)_ = 2.37, *p *=* *0.0382; Fig. 6*E*]. Although KO males also showed a trend toward reduced interaction, with a 14% decrease [M_diff_ = −14.59 s (95% CI, −28.54, 0.18); unbiased Cohen’s *d* = −0.52 (95% CI, −1.08, 0.00)], this difference is even smaller than for WT^MGH^ males and is unlikely to reflect a real change in behavior (Dunnett’s test, KO vs WT^SGH^: *q*_(1,72)_ = 2.07, *p *=* *0.0771). This result again points to an effect of housing on the social behavior of WT^MGH^ males.

In summary, adult KO males displayed a robust phenotype of reduced anxiety in the elevated plus maze test, and WT^MGH^ males showed altered behavior at P21, with increased activity during the first 5 min of the open field test and reduced social interaction.

**Figure 7.**
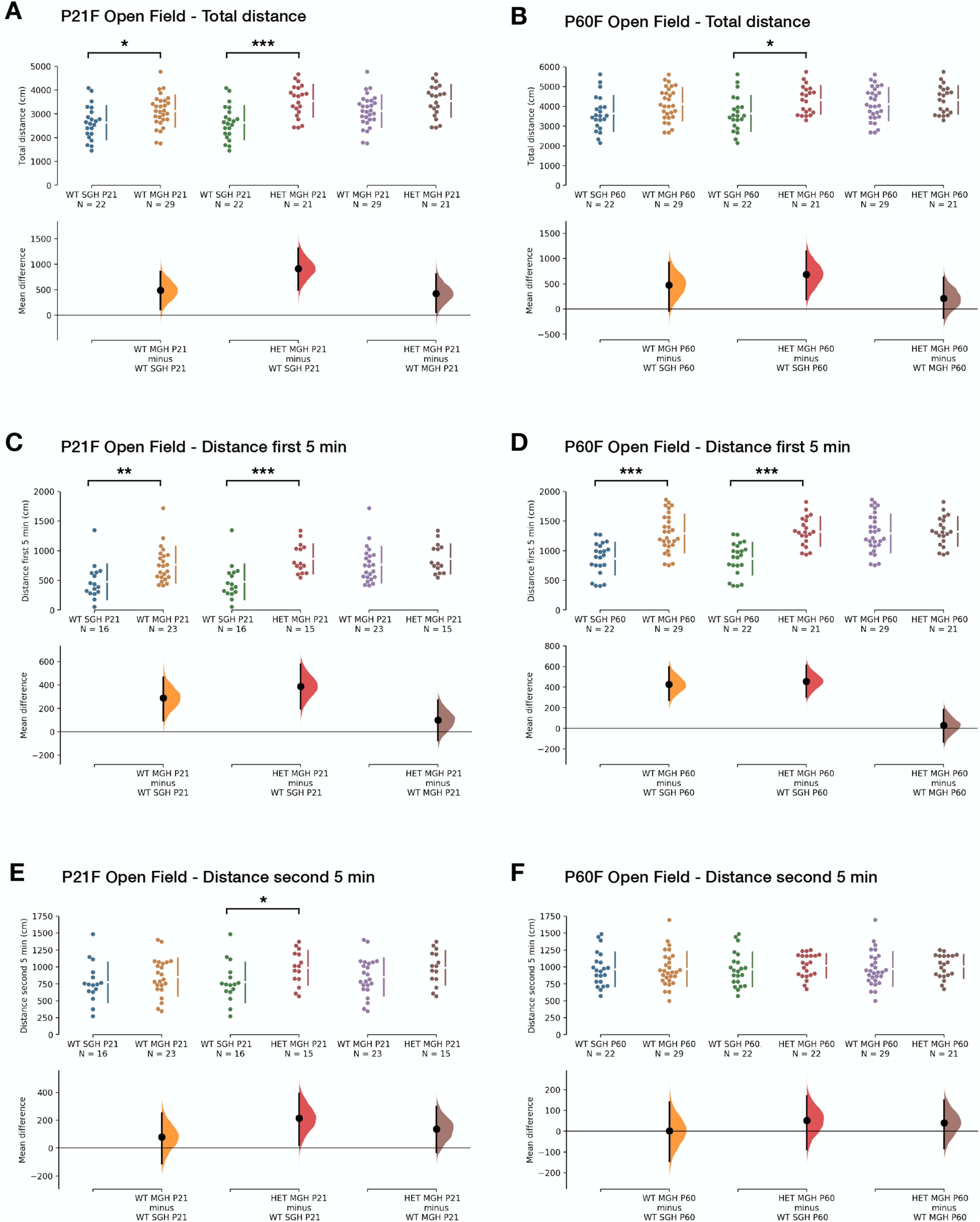
Behavioral alterations in the open field test in *Pcdh19* HET females and their WT littermates. ***A***, ***B***, Total distance traveled by females during the 20 min of the open field test at P21 (***A***) and P60 (***B***). ***C***, ***D***, Distance traveled by females during the first 5 min of the open field test at P21 (***C***) and P60 (***D***). ***E***, ***F***, Distance traveled by females during the second 5 min of the open field test at P21 (***E***) and P60 (***F***). Results correspond to the second day of testing at each age. For all panels, the top axis shows the raw data points for each group. To their right, the gap in the line indicates the mean, and the lines extending vertically represent the SD. The group and group sizes are indicated at the bottom. Note that each group appears twice in every graph, but with two different colors. The mean difference for each comparison is plotted in the lower axis as a bootstrap sampling distribution. The black dot represents the mean, and the vertical bar represents the 95% confidence interval. At the top of each graph the significance scores of the one-way ANOVA or Kruskal–Wallis test and their *post hoc* test are indicated. *p < 0.05; **p < 0.01; ***p < 0.001. Test results with female animals for the open field and 24 h activity that did not reach significance are presented in Extended Data Figure 7-1.

Changes in behavior were more pronounced in female mice than in their male counterparts (Table 6). We found again differences in the total distance traveled during the open field test at P21, with HET and WT^MGH^ females displaying increases of 35% and 19%, respectively, when compared with single-genotype housed controls [HET vs WT^SGH^: M_diff_ = 913.74 cm (95% CI, 494.07, 1314.30); unbiased Cohen’s *d *=* *1.29 (95% CI, 0.68, 2.04); WT^MGH^ vs WT^SGH^: M_diff_ = 486.76 cm (95% CI, 108.12, 853.27); unbiased Cohen’s *d *=* *0.69 (95% CI, 0.14, 1.31); one-way ANOVA: *F*_(2,69)_ = 9.54; *p *=* *0.0002; Tukey’s test, HET vs WT^SGH^: *q*_(1,69)_ = 6.17; *p *=* *0.0001; for WT^MGH^ vs WT^SGH^: *q*(1,69) = 3.55; *p *=* *0.0382; Fig. 7*A*]. Unlike in males, this effect was maintained at P60, but only in HET females, which traveled on average 19% greater distance than WT^SGH^ animals [M_diff_ = 682.77 cm (95% CI, 189.66, 1149.25); unbiased Cohen’s *d *=* *0.83 (95% CI, 0.23, 1.51); one-way ANOVA: *F*_(2,69)_ = 3.99; *p *=* *0.0229; Tukey’s test, HET vs WT^SGH^: *q*_(1,69)_ = 3.87; *p *=* *0.0214; Fig. 7*B*].

**Table 6:** Statistical analysis of the behavioral experiments in P21 and adult females

Behavioral test	Sex	Age	Normal data?	Equal variance?	Test	Results
Open field test: total distance day 2	F	P21	Yes	Yes	One-way ANOVA	*F*_(2,69)_ = 9.539; *p *=* *0.0002 *Post hoc* Tukey’s test: WT^SGH^ vs WT^MGH^; *p *=* *0.0382 WT^SGH^ vs HET; *p *=* *0.0001 WT^MGH^ vs HET; *p *=* *0.0837
Open field test: total distance day 2	F	P60	Yes	Yes	One-way ANOVA	*F*_(2,69)_ = 3.990; *p *=* *0.0229 *Post hoc* Tukey’s test: WT^SGH^ vs WT^MGH^; *p *=* *0.1094 WT^SGH^ vs HET; *p *=* *0.0214 WT^MGH^ vs HET; *p *=* *0.6459
Open field test: intervals day 2: 0–5 min	F	P21	No	Yes	Kruskal–Wallis	*H*_(2)_ = 21.86; *p *<* *0.0001 *Post hoc* Dunn’s test: WT^SGH^ vs WT^MGH^; *p *=* *0.0055 WT^SGH^ vs HET; *p *<* *0.0001 WT^MGH^ vs HET; *p *=* *0.2018
Open field test: intervals day 2: 5–10 min	F	P21	Yes	Yes	One-way ANOVA	*F*_(2,69)_ = 3.290; *p *=* *0.0432 *Post hoc* Tukey’s test: WT^SGH^ vs WT^MGH^; *p *=* *0.5888 WT^SGH^ vs HET; *p *=* *0.0359 WT^MGH^ vs HET; *p *=* *0.2036
Open field test: intervals day 2: 10–15 min	F	P21	Yes	Yes	One-way ANOVA	*F*_(2,69)_ = 2.102; *p *=* *0.13
Open field test: intervals day 2: 15–20 min	F	P21	Yes	Yes	One-way ANOVA	*F*_(2,69)_ = 1.038; *p *=* *0.36
Open field test: intervals day 2: 0–5 min	F	P60	Yes	Yes	One-way ANOVA	*F*_(2,69)_ = 17.95; *p *<* *0.0001 *Post hoc* Tukey’s test: WT^SGH^ vs WT^MGH^; *p *<* *0.0001 WT^SGH^ vs HET; *p *<* *0.0001 WT^MGH^ vs HET; *p *=* *0.9276
Open field test: intervals day 2: 5–10 min	F	P60	Yes	Yes	One-way ANOVA	*F*_(2,69)_ = 0.228; *p *=* *0.797
Open field test: intervals day 2: 10–15 min	F	P60	Yes	Yes	One-way ANOVA	*F*_(2,69)_ = 1.068; *p *=* *0.349
Open field test: intervals day 2: 15–20 min	F	P60	No	Yes	Kruska–Wallis	*H*_(2)_ = 3.2334; *p *=* *0.1986
Open field test: time in left day 2	F	P21	No	Yes	Kruska–Wallis	*H*_(2)_ = 4.6819; *p *=* *0.0962
Open field test: time in left day 2	F	P60	No	Yes	Kruska–Wallis	*H*_(2)_ = 4.0863; *p *=* *0.1296
24 h activity: total	F	>P60	Yes	Yes	One-way ANOVA	*F*_(2,36)_ = 1.077; *p *=* *0.3512
24 h activity: light period	F	>P60	Yes	Yes	One-way ANOVA	*F*_(2,36)_ = 2.290; *p *=* *0.1159
24 h activity: dark period	F	>P60	Yes	Yes	One-way ANOVA	*F*_(2,36)_ = 1.103; *p *=* *0.3429
Elevated plus maze	F	P21	No	Yes	Kruskal–Wallis	*H*_(2)_ = 20.943; *p *<* *0.001 *Post hoc* Dunn’s test: WT^SGH^ vs WT^MGH^; *p *=* *0.8101 WT^SGH^ vs HET; *p *=* *0.0042 WT^MGH^ vs HET; *p *<* *0.0001
Elevated plus maze	F	P60	Yes	Yes	One-way ANOVA	*F*_(2,69)_ = 5.085; *p *=* *0.0041 *Post hoc* Tukey’s test: WT^SGH^ vs WT^MGH^; *p *=* *0.5689 WT^SGH^ vs HET; *p *=* *0.0043 WT^MGH^ vs HET; *p *=* *0.0401
Social interaction	F	P21	Yes	Yes	One-way ANOVA	*F*_(2,69)_ = 1.297; *p *=* *0.2425
Social interaction	F	P60	Yes	Yes	One-way ANOVA	*F*_(2,69)_ = 3.536; *p *=* *0.0398 *Post hoc* Dunnett’s test (all vs WT SGH): WT^SGH^ vs WT^MGH^; *p *=* *0.0432 WT^MGH^ vs HET; *p *=* *0.9654

The table includes the behavioral test analyzed, sex and age of the animals, data and variance distribution, statistical test used, and results obtained. F, female.

10.1523/ENEURO.0510-20.2021.f7-1 Figure 7-1 ***A***, ***B***, Distance travelled in the last two 5 min intervals of the open field test by P21 (***A***) and P60 (***B***) females. ***C***, Number of beam breaks during the 24 h activity test for the females of the different conditions. Light, Light phase; dark, dark phase. ***D***, Number of beam breaks by hour in the 24 h activity test for the females of the different conditions. The time of the day is shown on the *x*-axis, and the gray square indicates the hours of the dark period. ***E***, ***F***, Time spent by females in the center of the arena during the 20 min open field test at P21 (***E***) and P60 (***F***). In these two panels, raw data are depicted in the top axis, with the mean (gap) and SD (vertical bars) to their right. Group and group sizes are indicated at the bottom. Note that each group appears twice in every graph, but in two different colors. The mean difference for each comparison is plotted in the lower axis as a bootstrap sampling distribution. The mean is indicated by the black dot, and the 95% CI by the vertical bars. The numbers of tested animals were as follows: at P21 and P60: WT^SGH^, 22; WT^MGH^, 29; HET, 21. For the 24 h activity test, numbers were as follows: WTSGH, 18; WTMGH, 11; HET, 10. Results are indicated as the mean ± SEM. *p < 0.05. Download Figure 7-1, TIF file.

Analysis by 5 min intervals showed that the increase in total distance was mainly because of increased activity during the first 5 min in the open field arena both at preweaning age and in adults (Fig. 7*C*,*D*). This effect was strong at both ages for HET females and their WT siblings when compared with single-genotype housed females, with increases of 95% (HET) and 54% (WT^MGH^) at P21, and 53% (HET) and 49% (WT^MGH^) in adult animals. At P21, the M_diff_ between HET and WT^SGH^ was 388.61 cm (95% CI, 195.54, 576.41) with an unbiased Cohen’s *d *=* *1.49 (95% CI, 0.87, 2.27). Between WT^MGH^ and WT^SGH^, M_diff_ = 289.11 cm (95% CI, 94.48, 465.99) with an unbiased Cohen’s *d *=* *0.96 (95% CI, 0.40, 1.61; Kruskal–Wallis test: *H*_(2)_ = 21.86; *p *<* *0.0001; Dunn’s test, HET vs WT^SGH^: *z *=* *4.61; *p *<* *0.0001; WT^MGH^ vs WT^SGH^: *z *=* *3.12; *p *=* *0.0055; Fig. 7*C*). Despite smaller percentage increases, the M_diff_ values between HET and WT^SGH^, and WT^MGH^ and WT^SGH^ at P60 rose to 456.75 cm (95% CI, 304.66, 609.57) and 426.36 cm (95% CI, 271.11, 595.22), respectively. The unbiased Cohen’s *d* values for those comparisons were 1.73 (95% CI, 1.09, 2.55) and 1.39 (95% CI, 0.82, 2.09; one-way ANOVA: *F*_(2,69)_ = 17.95; *p *<* *0.0001; Tukey’s test, HET vs WT^SGH^: *q*_(1,69)_ = 7.38; *p *<* *0.0001; WT^MGH^ vs WT^SGH^: *q*_(1,69)_ = 7.43; *p *<* *0.0001; Fig. 7*D*). HET females also traveled a 25% longer distance than WT^SGH^ females during the second 5 min interval at P21 [M_diff_ = 215.18 cm (95% CI, 20.06, 391.89); unbiased Cohen’s *d *=* *0.85 (95% CI, 0.25, 1.54); one-way ANOVA: *F*_(2,69)_ = 3.29; *p *=* *0.0432; Tukey’s test, HET vs WT^SGH^: *q*_(1,69)_ = 3.58; *p *=* *0.0359; Fig. 7*E*], suggesting a potential effect of genotype in addition to the housing effect. By P60, though, there was no average change between the distance run in the second 5 min by any of the groups (Fig. 7*F*), and no other differences were apparent during the rest of the testing period (Extended Data Fig. 7-1*A*,*B*). Similar to male mice, the spontaneous activity over 24 h, measured as the number of beam breaks, was not altered for any of the three experimental groups in the light (one-way ANOVA, main effect of genotype: *F*_(2,36)_ = 2.29; *p *=* *0.1159), dark (one-way ANOVA, main effect of genotype: *F*_(2,36)_ = 1.10; *p *=* *0.3429), or total periods (one-way ANOVA, main effect of genotype: *F*_(2,36)_ = 1.08; *p *=* *0.3512; Extended Data Fig. 7-1*C*,*D*). Again, isolated differences were evident at two time points during the dark phase (10:00 P.M.: one-way ANOVA, main effect of genotype: *F*_(2,36)_ = 3.84; *p *= 0.0309; Tukey’s test, WT^MGH^ vs WT^SGH^, *q*_(1,69)_ = 3.65; *p *=* *0.0364; 4:00 A.M.: Welch’s ANOVA, *W*_(2,23.61)_ = 8.52; *p *=* *0.0016; Dunnett’s T3, WT^MGH^ vs HET: *t*_(2,18.32)_ = 3.83; *p *= 0.0036; Dunnett’s T3, WT^MHG^ vs WT^SGH^: *t*_(2,23.41)_ = 2.65; *p *=* *0.0417; Extended Data Fig. 7-1*D*), but no overall changes in activity were apparent in this test.

**Figure 8.**
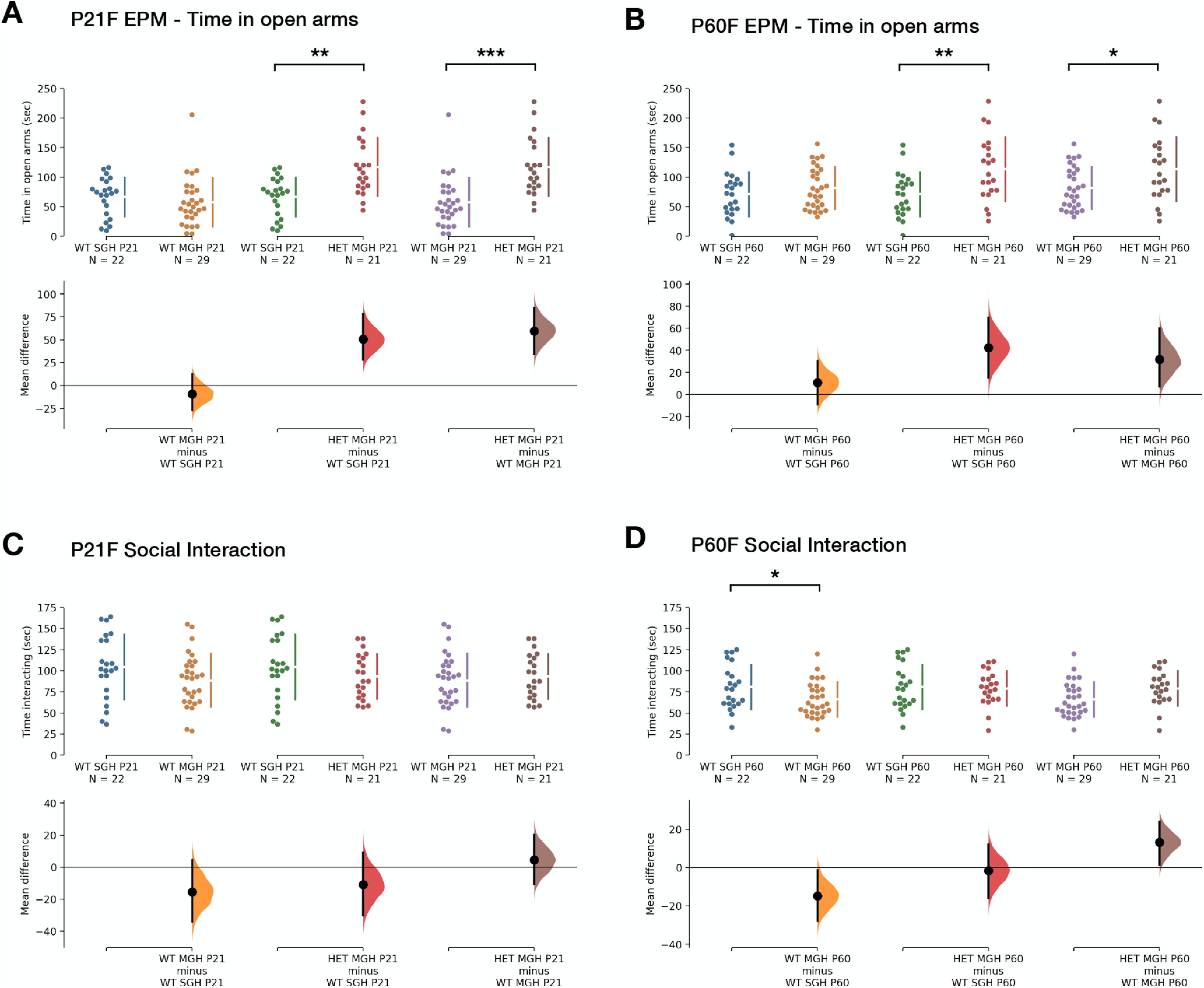
Behavioral alterations in the EPM and social interaction tests in *Pcdh19* HET females and their WT littermates. ***A***, ***B***, Total time spent by females in the open arms of the elevated plus maze during the 5 min test at P21 (***A***) and P60 (***B***). ***C***, ***D***, Time spent by females interacting with a nonfamiliar female at P21 (***C***) and P60 (***D***). The total duration of the test was 5 min. For all panels, the top axis shows the raw data points for each group. To their right, the gap in the line indicates the mean, and the lines extending vertically represent the SD. The group and group sizes are indicated at the bottom. Note that each group appears twice in every graph, but with two different colors. The mean difference for each comparison is plotted in the lower axis as a bootstrap sampling distribution. The black dot represents the mean, and the vertical bar represents the 95% confidence interval. At the top of each graph the significance scores of the one-way ANOVA or Kruskal–Wallis test and their *post hoc* test are indicated. *p < 0.05; **p < 0.01; ***p < 0.001.

Since the increase in distance traveled during the first 5 min in the open field test does not seem to be caused by the overall hyperactivity of HET animals and their WT siblings, we again analyzed anxiety-related behaviors in these animals. There were no differences in the time spent in the center of the open field arena for any of the conditions at P21 (Kruskal–Wallis test: *H*_(2)_ = 4.68; *p *= 0.0962) or P60 (Kruskal–Wallis test: *H*_(2)_ = 4.09; *p *=* *0.1296; Extended Data Fig. 7-1*E*,*F*), but, similar to the results obtained with male animals, HET females spent considerably more time in the open arms of the elevated plus maze than any of the WT females at P21 and P60 (Fig. 8*A*,*B*). The increases against WT^SGH^ and WT^MGH^ animals amounted to 76% and 103% at preweaning age [HET vs WT^SGH^: M_diff_ = 50.69 s (95% CI, 28.24, 78.00); unbiased Cohen’s *d *=* *1.20 (95% CI, 0.59, 1.94); HET vs WT^MGH^: M_diff_ = 59.71 s (95% CI, 34.28, 84.71); unbiased Cohen’s *d *=* *1.32 (95% CI, 0.74, 2.02); Kruskal–Wallis test: *H*_(2)_ = 20.94, *p *<* *0.0001; Dunn’s test, HET vs WT^SGH^: *z *=* *3.19, *p *=* *0.042; WT^MGH^ vs WT^SGH^: *z *=* *4.49, *p *< 0.0001]. In adults, the increase was down to 60% and 39% [HET vs WT^SGH^: M_diff_ = 42.40 s (95% CI, 15.09, 69.81); unbiased Cohen’s *d *=* *0.90 (95% CI, 0.30, 1.60); HET vs WT^MGH^: M_diff_ = 31.69 s (95% CI, 7.28, 59.92); unbiased Cohen’s *d *=* *0.71 (95% CI, 0.15, 1.34); one-way ANOVA: *F*_(2,69)_ = 5.95; *p *=* *0.0041; Tukey’s test, HET vs WT^SGH^: *q*_(1,69)_ = 4.67; *p *=* *0.0043; HET vs WT^MGH^: *q*_(1,69)_ = 3.72; *p *=* *0.0281]. These results indicate a strong effect of genotype on reducing anxiety, as also seen for adult male KO animals.

As in the case of male mice, the social interaction test revealed differences between single-genotype and mixed-genotype housed WT females (Fig. 8*C*,*D*). However, this effect was present only in adult animals, with WT^MGH^ females spending 15% less time interacting with an unfamiliar female in estrus [M_diff_ = −14.69 s (95% CI, −27.79, −1.29); unbiased Cohen’s *d* = −0.62 (95% CI, −1.24, −0.07); one-way ANOVA: *F*_(2,69)_ = 3.38; *p *=* *0.0398; Dunnett’s test, WT^MHG^ vs WT^SGH^: *q*_(1,69)_ = 2.32, *p *=* *0.0432].

Overall, we found significant behavioral differences between wild-type and mutant animals that were generally more pronounced in HET females than in KO males. HET females displayed consistent hyperactivity during the first 5 min of the open field test and, similar to the mutant males, a robust phenotype of decreased anxiety in the elevated plus maze, in this case both at preweaning and at adult stages. Importantly, we also uncovered an effect of housing on the behavior of WT animals, with WT^MGH^ males and females presenting significant differences in the open field and social interaction tests when compared with single-genotype housed WT animals.

## Discussion

Recent studies have shed light on the different functions of PCDH19 (Pederick et al., 2016, 2018; Hayashi et al., 2017; Pham et al., 2017; Bassani et al., 2018; Homan et al., 2018; Serratto et al., 2020; Hoshina et al., 2021; Mincheva-Tasheva et al., 2021; for review, see Gerosa et al., 2019; Gecz and Thomas, 2020), but we still have limited knowledge about the neuronal types expressing PCDH19 and the consequences of *Pcdh19* mutations on fine cortical composition, despite the relevance of these factors to understand the pathologic mechanisms underpinning EIEE9. Here we present a detailed analysis of neuronal subtypes expressing *Pcdh19* in the mouse somatosensory cortex and a comparison with human data. Our study reveals that *Pcdh19*/*PCDH19* is not only expressed in pyramidal neurons, but also in different types of interneurons, and that, in general, higher expression is limited to specific subpopulations in both cases. Our analysis also rules out major anomalies in the main axonal tracts and provides a quantitative assessment of cortical composition and lamination. Despite the lack of major architectural defects, our data reveal subtle defects in layer composition that could contribute to the pathophysiology of EIEE9. Indeed, mutant animals display behavioral alterations in the open field test (females) and elevated plus maze test (males and females). Importantly, and as previously revealed with the analysis of *Nlgn3* mutants (Kalbassi et al., 2017), the *Pcdh19* mutation affects the behavior of wild-type littermates when housed in the same cage.

Hitherto, the characterization of the neuronal populations expressing PCDH19 has been hindered by the lack of specific antibodies that perform satisfactorily in immunohistochemistry analyses. In addition, as PCDH19 is likely distributed in both axons and dendrites (Pederick et al., 2016; Hayashi et al., 2017; Bassani et al., 2018), the unambiguous identification of cell bodies expressing PCDH19 is a challenging objective, as is the case for most membrane proteins in the cortex. To overcome this difficulty, we focused on the expression of *Pcdh19* mRNA, which is detected in the cell soma and allows a better assessment of coexpression with other neuronal markers, which tend to be either nuclear or cytoplasmic. Although mRNA and protein expression are not necessarily correlated, available data show a good match between the regions with the strongest mRNA and protein signals (Hayashi et al., 2017; Pederick et al., 2018). Our ISH/IHC combination approach provides experimental evidence for the expression of *Pcdh19* by different neuronal types across cortical layers, including interneurons. We chose the somatosensory cortex to carry out the analysis because it is a very well characterized area with a good definition of cortical layers. We then confirmed the results obtained in the postnatal sSC by choosing single-cell RNA sequencing (scRNAseq) datasets that include neurons from various cortical regions (including sSC) from adult brain, which allowed us to obtain a global view of *Pcdh19*/*PCDH19* expression across cortical areas in mouse and human.

Our analysis of a mouse dataset of whole cortex and hippocampus confirmed that *Pcdh19* is expressed by excitatory neurons in layer V, projecting both intracortically and extracortically, as well as by certain subtypes of layer II/III projection neurons, in agreement with the ISH data. Expression in layer IV is harder to judge from the scRNAseq results, as there are no clusters representing neurons from layer IV exclusively, but several clusters in layers VI and VIb also show high *Pcdh19* expression. In interneurons, the expression is widespread in the Pvalb subclass; cluster specific in the Sncg, Vip, and Sst subclasses; and very low in the Lamp5 and Pax6 clusters, except for Lamp5 Lhx6, which shows high expression. These results demonstrate that while *Pcdh19* is expressed by a variety of excitatory and inhibitory neurons, expression remains specific for particular clusters. This cluster specificity would suggest a role for PCDH19 in the establishment of neuronal circuits as a potential neuronal recognition molecule.

Human *PCDH19* follows a similar pattern, with expression in both excitatory and inhibitory neuronal types. Expression in human excitatory neurons of the sSC is more graded, with many more subtypes showing intermediate expression levels than in mouse, likely reflecting an averaging effect because of the smaller number of human clusters defined for that dataset. In any case, highest expression corresponds to clusters in layers III and V, in line with RNA ISH results in mice. Regarding interneurons, high *PCDH19* expression can be found in subtypes of LAMP5, VIP, SST, and PVALB interneurons, which generally show a good correlation with their murine counterparts. This is a relevant finding that supports the use of mouse models to investigate some aspects of *PCDH19* GCE. However, it is important to note that there are some differences as well, like the comparatively lower expression of *PCDH19* in long-range projecting interneurons in humans (Inh L6 SST NPY in human, Sst_Chodl in mouse). The functional relevance of *Pcdh19*/*PCDH19* expression in particular neuronal subtypes will need to be established experimentally, but our results provide a framework to support those functional studies in the future, not least because of regional differences in the expression of this gene within neuronal subtypes.

To date, no detailed quantitative characterization of cortical composition and lamination has been performed in the three existing *Pcdh19* KO models (Pederick et al., 2016; Hayashi et al., 2017; Hoshina et al., 2021). We have quantified five excitatory and four inhibitory markers, looking at overall abundance, as well as distribution throughout the cortical plate in the somatosensory cortex. Our analysis, which was conducted separately in males and females, reveals no differences in the abundance of the different excitatory neuronal types analyzed, but points to small decreases in somatostatin-expressing interneurons in HET females and calretinin^+^ cells in KO males. We confirm the lack of major lamination defects (Pederick et al., 2016; Hayashi et al., 2017; Hoshina et al., 2021); however, our quantitative approach exposes more subtle changes in the distribution of certain neuronal types, indicating altered composition of specific layers or sublayers. Although some changes might represent false-positive findings, such as the ones for HET Pvalb bin 8 and KO CR bin 7, which might be explained by abnormal distributions that were apparent in the comparison between WT males and females, it is worth noting that changes between genotypes were more frequent and, in many cases, more significant than between WT animals of opposite sex. Indeed, we did not find a single difference between WT males and females at P10, suggesting that, although subtle, changes in layer composition cannot be ruled out in *Pcdh19* mutants. Given the degree of neuronal diversity revealed by recent scRNAseq studies, our results also support the possibility of more widespread differences affecting other neuronal subtypes not covered by the antibodies used in our analysis. The origin of these differences is unknown, but one possibility is that they could arise as a consequence of altered neurogenesis, since PCDH19 has been shown to play a role in this process (Fujitani et al., 2017; Homan et al., 2018; Lv et al., 2019). It is also important to consider that we conducted our analysis mainly in the sSC, but, given that *Pcdh19* expression varies between cortical regions, it is possible that different areas might be affected in different ways by a total or partial loss of PCDH19. Reports of focal cortical dysplasia and limbic abnormalities in EIEE9 patients (Kurian et al., 2018; Pederick et al., 2018; Lenge et al., 2020) and focal areas of disorganization in ASD patients (Stoner et al., 2014) seem to support this possibility.

Despite the involvement of other δ-protocadherins in the development of axonal tracts (Uemura et al., 2007; Piper et al., 2008; Biswas et al., 2014; Hayashi et al., 2014), our data do not support a major role of PCDH19 in this process. We did not detect any alterations in the main axonal tracts in the brain after staining for the axonal protein L1CAM, and a more detailed analysis of the corpus callosum also showed no differences in its dorsoventral extension or the dorsal restriction of Neuropilin-1-expressing axons. This is in agreement with the lack of defects found by Hayashi et al. (2017) in the projection of axons through this particular tract. More subtle defects in specific tracts would require much deeper analyses to be revealed, as the defects in cortical axonal arborization recently described in *Pcdh19* HET animals (Mincheva-Tasheva et al., 2021).

Regardless of any anatomic alterations, investigating behavior allows a relevant functional assessment of the consequences of *Pcdh19* loss. Our analysis differed from those in previous studies (Hayashi et al., 2017; Lim et al., 2019) in two main ways. First, in addition to adult animals, we also tested animals at a much younger age (preweaning, P21), as EIEE9 is a developmental disorder and therefore it is relevant to determine when any behavioral changes begin. Second, we added a second cohort of control animals: wild-type single-genotype housed mice, which have only been exposed to other WT animals during their life. An effect of WT littermates on the behavior of mutant animals was shown by Yang et al. (2011), when they demonstrated that raising less sociable BTBR T+tf/J mice with highly sociable C57BL6/J animals improved BTBR T+tf/J sociability. However, the impact of social environment on the behavior of WT littermates has only recently been demonstrated in a study with mice mutant for *Nlgn3*, an X-linked cell adhesion protein that has been implicated in ASD (Kalbassi et al., 2017). Therefore, this is further evidence to suggest that mutant mice can alter the behavior of their WT littermates and to support the addition of single-genotype housed WT controls.

In line with a previous mouse study (Hayashi et al., 2017) and with the findings in human patients, changes in behavior were more apparent in HET females than in their KO male siblings. *Pcdh19* KO males only showed increased time spent in the open arms of the EPM, indicating reduced anxiety, when tested as adults. This same behavior was displayed by young *Pcdh19* HET females (P21), which maintained it into adulthood. However, HET females also exhibited increased exploratory behavior, or maybe hypersensitivity to new environments, from a young age, as demonstrated by their consistently higher distance traveled during the first 5 min in the open field test at P21 and P60. It is important to consider that animals were placed into the open field arena four times in total, as they were tested on 2 consecutive days at both ages. Although habituation to the environment would be expected in this situation, the increased distance traveled during the first 5 min was apparent in all four trials, indicating a robust behavioral response. These results also suggest that behavioral changes in *Pcdh19* heterozygous animals start early in life, validating them as a good model for a developmental condition.

Open field and EPM tests were also performed in the study by Hayashi et al. (2017). They found no differences in the EPM test, but this could be because of differences in experimental design or in the mouse model used for the test. Regarding the open field test, Hayashi et al. (2017) found no differences in total distance or time in the center when the test was conducted at 11–12 weeks of age. However, when they repeated the test 23 weeks later, *Pcdh19* HET females spent significantly more time in the center of the open field arena, suggesting reduced anxiety. Although our animals did not display such behavior, they were tested at approximately P60, which would be in agreement with the data from their first open field test. In addition, the results of our EPM test also indicate reduced anxiety in our animals, which could therefore represent a behavioral characteristic of *Pcdh19* mutant animals. Because no specific analysis of the first 5 min was conducted in that study, it is difficult to assess whether their animals exhibited increased activity during that period. Nevertheless, the fact that WT females display the same behavioral phenotype as their HET siblings indicates an effect of the social environment that can only be detected through the inclusion of single-genotype housed WT animals. Interestingly, this effect was also present in young males, with WT^MGH^ animals traveling a higher distance in the first 5 min of the open field test than KO or WT^SGH^ males. However, unlike in the female population, this behavior disappeared in adulthood. Because adult male and female animals are housed separately, it is tempting to speculate that this effect of the social environment is somehow mediated by the HET females, although other causes, like a maternal effect, cannot be ruled out based on our experiments.

One of the comorbidities of EIEE9 patients is ASD (Kolc et al., 2020), and changes in *PCDH19* have also been linked to ASD cases (Piton et al., 2011; van Harssel et al., 2013). Indeed, a recent behavioral study with the Taconic Biosciences *Pcdh19* KO mouse model has revealed social interaction deficits in the three-chamber test in KO males and HET females, as well as increased repetitive behavior in males (females were not tested; Lim et al., 2019). In our analysis, we also found differences in social behavior, but, interestingly, only in WT^MGH^ animals. Both males and females spent less time interacting with a stranger female at P21 and P60, respectively, than with WT^SGH^ animals, in what appears to be another example of the effect of the environment on mouse behavior. Since males were not tested at P60, because at that age it becomes a measure of courtship behavior rather than simple social interaction and as such is not comparable to the P21 behavior, we do not know whether this phenotype would be maintained into adulthood or whether, similar to the results of the open field test, it would revert to normal with age. The fact that HET and KO animals did not differ in their behavior from their WT littermates is in contradiction with the results from the study by Lim et al. (2019), although different tests were conducted in the two studies, making a direct comparison difficult. In summary, our behavioral characterization of the *Pcdh19* Taconic Biosciences mouse model reveals a stronger effect of *Pcdh19* mutation in HET females than in KO males and a significant effect of the social environment on the behavior of WT littermates, as previously described for *Nlgn3* mutant animals (Kalbassi et al., 2017). This is a relevant finding, and this effect should be taken into consideration for the design of future behavioral experiments, as the failure to do so could result in the misinterpretation of data and missed behavioral phenotypes. It is important to note that, despite the subtle differences found in cortical composition in the sSC, we believe that a correlation between those changes and the observed behavioral alterations cannot be made at this point. Different cortical and brain regions are involved in the control of the behavioral paradigms that we have analyzed, so isolated cellular results of one cortical area, however widespread they might be, cannot be linked to any specific aspects of behavior. Such a correlation would require functional assays of neuronal function to go beyond mere speculation.

Finally, an important question is why the mutation of *Pcdh19* in mice leads to much milder defects than in humans, with the absence of seizures as the most striking difference. It is worth noting that similar results have been described for other neurodevelopmental disorders that present with epilepsy, such as CDKL5 deficiency disorder or fragile X syndrome (FXS). Mice carrying either a null allele for *Cdkl5* or the disease-causing mutation R59X do not display behavioral seizures, but they exhibit network hyperexcitability that manifests as decreased threshold to pharmacologically induced seizures (Wang et al., 2012; Amendola et al., 2014). In the case of FXS, in which epilepsy develops in ∼20% of patients (Musumeci et al., 1999; Sabaratnam et al., 2001), none of the KO mouse models presents spontaneous seizures. However, they are susceptible to audiogenic seizures and display alterations in cortical EEG frequency (Musumeci et al., 2000; Lovelace et al., 2018; Goswami et al., 2019). Similarly, cortical network activity is altered in *Pcdh19* heterozygous animals (Pederick et al., 2018), indicating that mutations in those genes in mice do alter cortical connectivity, but not enough to trigger seizures. The smaller size and reduced complexity of the mouse brain probably account, at least partially, for these discrepancies, maybe by conferring a generally lower susceptibility to seizures in mice. Therefore, considering recent progress in the use of brain organoids for the study of neuronal connectivity (Quadrato et al., 2017), this emerging model might be needed in the future to dissect the effects of *PCDH19* mutations on human connectivity.

## Synthesis

Reviewing Editor: Alfonso Represa, INSERM Decisions are customarily a result of the Reviewing Editor and the peer reviewers coming together and discussing their recommendations until a consensus is reached. When revisions are invited, a fact-based synthesis statement explaining their decision and outlining what is needed to prepare a revision will be listed below. The following reviewer(s) agreed to reveal their identity: Shinji Hirano.

Dear Authors,

With reviewer we appreciate the changes that were performed in the manuscript that solved some of the concerns evoked in the previous review. It was evoked there that 'histological” and “behavioral” data, were not necessarily correlated but we understand that little can be done to clarify this issue. However, we agree that cellular data will be of interest for readers in the field and that behavioral data, particularly the differences related to housing conditions, will be important for a wider audience.

The reorganization of data focusing mainly now on somatosensory cortex, contributes to clarify the first part of the manuscript.

In response to point 4, gender data provided indicates some differences. Though the phenotype is subtle to make clear conclusions, we think they highlight the interest for taking gender issues into consideration.

Concerning point 5, we understand the technical difficulties in the analysis of arborization of the cortical neurons and withdraw our previous comment on this.
