# Supplementary figures and images for: Cellular and Behavioral Characterization of Pcdh19 Mutant Mice: subtle Molecular Changes, Increased Exploratory Behavior and an Impact of Social Environment
Source: eNeuro. 2021 Aug 10;8(4):ENEURO.0510-20.2021. doi: 10.1523/ENEURO.0510-20.2021 (PMC8362684; doi:10.1523/ENEURO.0510-20.2021)

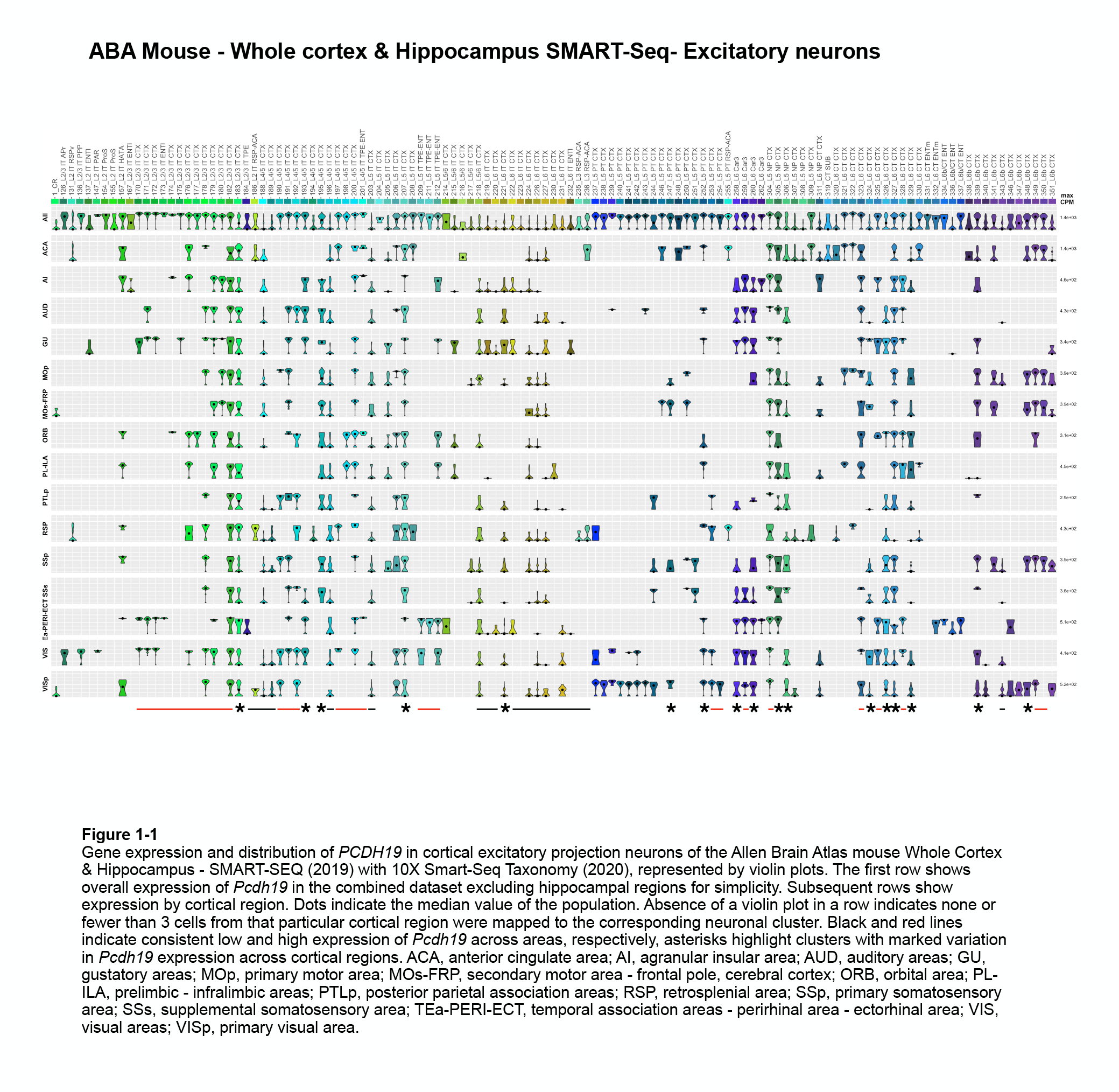

Supplement: Figure 1-1 — Gene expression and distribution of Pcdh19 in cortical excitatory projection neurons of the Allen Brain Atlas Mouse Whole Cortex & Hippocampus - SMART-SEQ (2019) with 10x Smart-Seq Taxonomy (2020), represented by violin plots. The first row shows the overall expression of Pcdh19 in the combined dataset excluding hippocampal regions for simplicity. Subsequent rows show expression by cortical region. Dots indicate the median value of the population. Absence of a violin plot in a row indicates that none or fewer than three cells from that particular cortical region were mapped to the corresponding neuronal cluster. Black and red lines indicate consistent low and high expression of Pcdh19 across areas, respectively; asterisks highlight clusters with marked variation in Pcdh19 expression across cortical regions. ACA, Anterior cingulate area; AI, agranular insular area; AUD, auditory areas; GU, gustatory areas; MOp, primary motor area; MOs-FRP, secondary motor area-frontal pole, cerebral cortex; ORB, orbital area; PL-ILA, prelimbic - infralimbic areas; PTLp, posterior parietal association areas; RSP, retrosplenial area; TEa-PERI-ECT, temporal association areas-perirhinal area-ectorhinal area; VIS, visual areas; VISp, primary visual area. Download Figure 1-1, TIF file. [file enu-eN-NWR-0510-20-s02.tif]

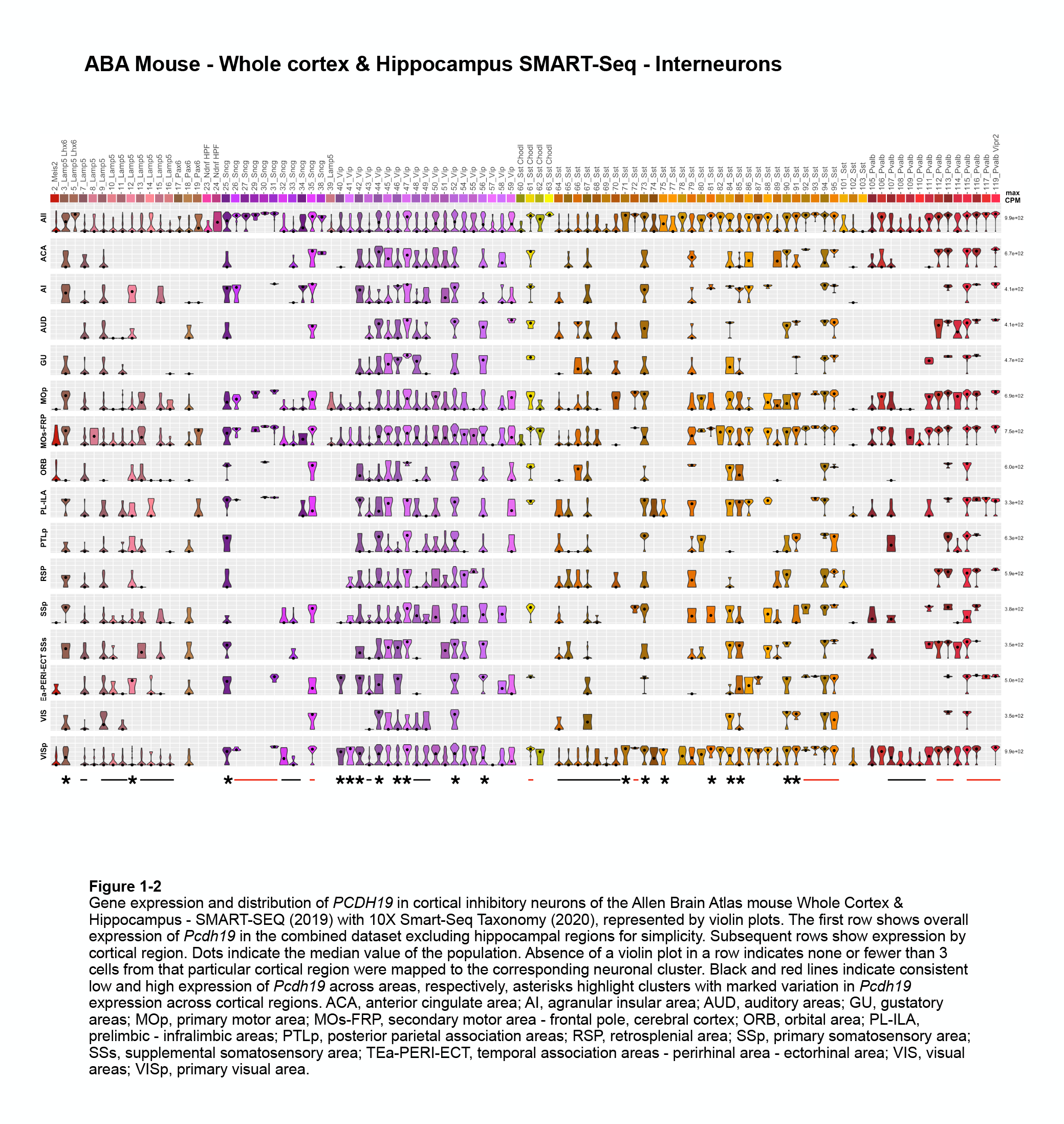

Supplement: Figure 1-2 — Gene expression and distribution of Pcdh19 in cortical inhibitory neurons of the Allen Brain Atlas Mouse Whole Cortex & Hippocampus - SMART-SEQ (2019) with 10x Smart-Seq Taxonomy (2020), represented by violin plots. The first row shows the overall expression of Pcdh19 in the combined dataset excluding hippocampal regions, for simplicity. Subsequent rows show expression by cortical region. Dots indicate the median value of the population. Absence of a violin plot in a row indicates that none or fewer than three cells from that particular cortical region were mapped to the corresponding neuronal cluster. Black and red lines indicate consistent low and high expression of Pcdh19 across areas, respectively; asterisks highlight clusters with marked variation in Pcdh19 expression across cortical regions. ACA, Anterior cingulate area; AI, agranular insular area; AUD, auditory areas; GU, gustatory areas; MOp, primary motor area; MOs-FRP, secondary motor area-frontal pole, cerebral cortex; ORB, orbital area; PL-ILA, prelimbic-infralimbic areas; PTLp, posterior parietal association areas; RSP, retrosplenial area; TEa-PERI-ECT, temporal association areas-perirhinal area-ectorhinal area; VIS, visual areas; VISp, primary visual area. Download Figure 1-2, TIF file. [file enu-eN-NWR-0510-20-s03.tif]

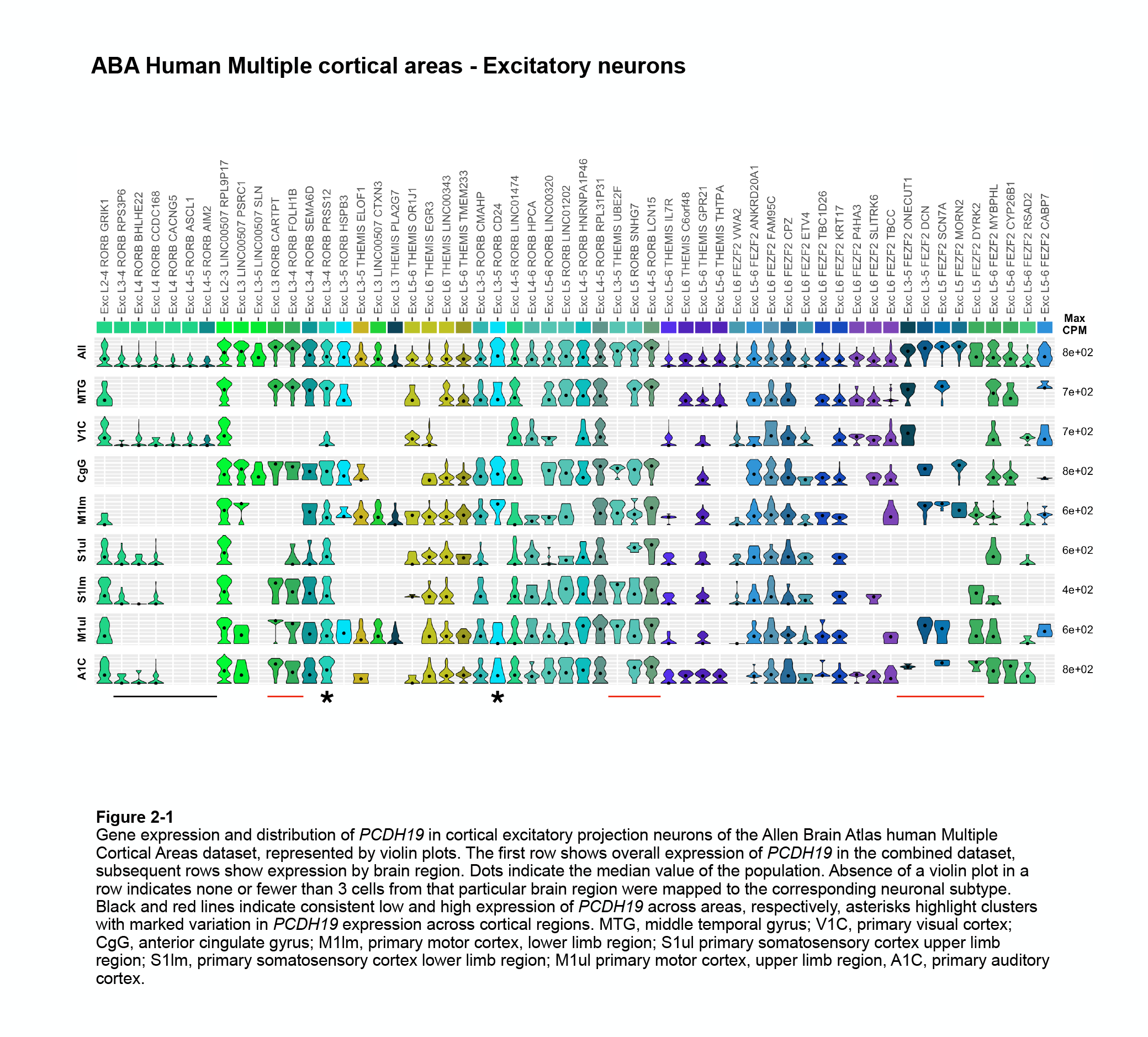

Supplement: Figure 2-1 — Gene expression and distribution of PCDH19 in cortical excitatory projection neurons of the Allen Brain Atlas Human Multiple Cortical Areas dataset, represented by violin plots. The first row shows overall expression of PCDH19 in the combined dataset; subsequent rows show expression by brain region. Dots indicate the median value of the population. Absence of a violin plot in a row indicates that none or fewer than three cells from that particular brain region were mapped to the corresponding neuronal subtype. Black and red lines indicate consistent low and high expression of PCDH19 across areas, respectively; asterisks highlight clusters with marked variation in PCDH19 expression across cortical regions. CgG, Anterior cingulate gyrus; M1lm, primary motor cortex, lower limb region; M1ul primary motor cortex, upper limb region, A1C, primary auditory cortex. Download Figure 2-1, TIF file. [file enu-eN-NWR-0510-20-s04.tif]

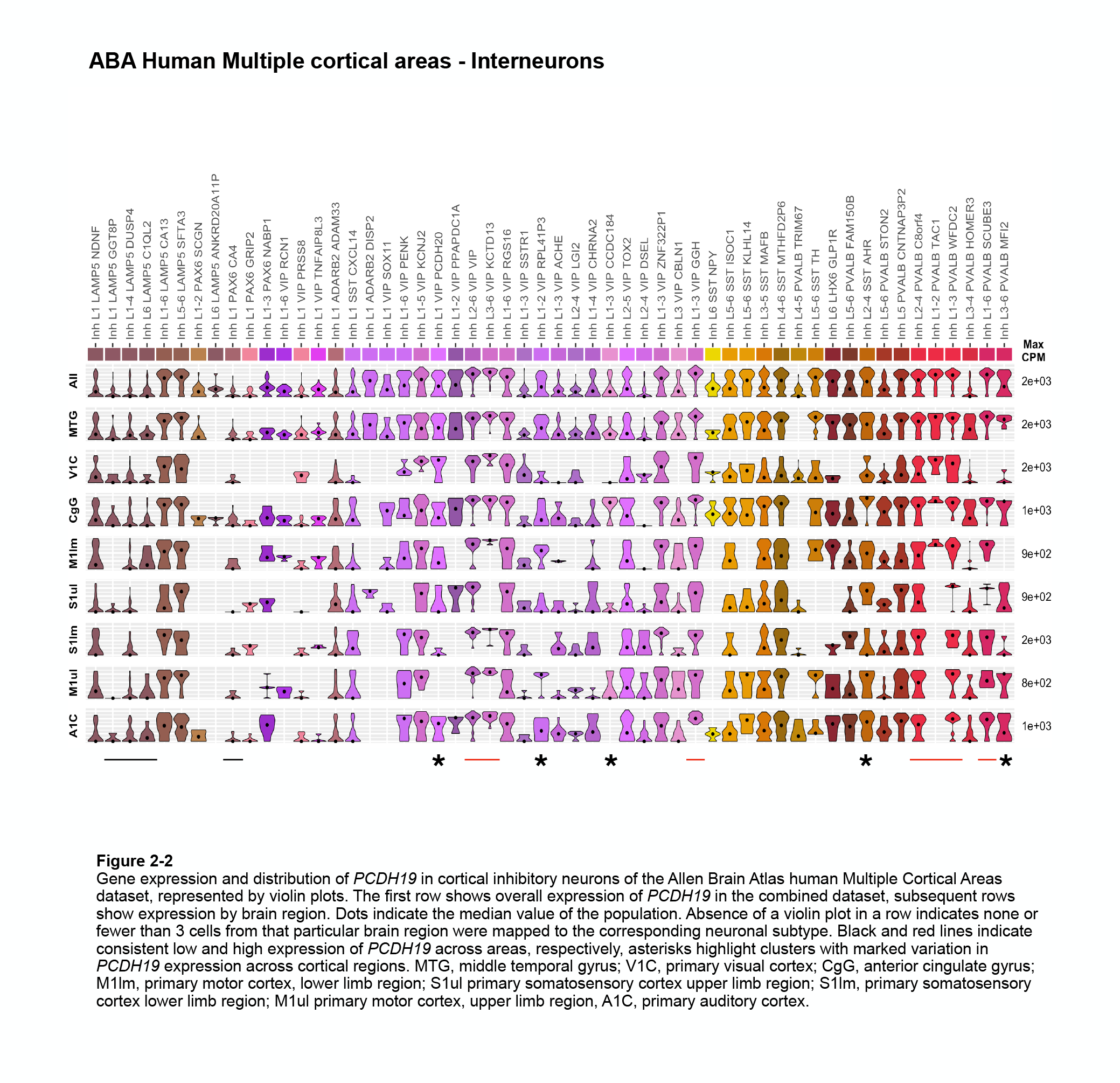

Supplement: Figure 2-2 — Gene expression and distribution of PCDH19 in cortical inhibitory neurons of the Allen Brain Atlas Human Multiple Cortical Areas dataset, represented by violin plots. The first row shows the overall expression of PCDH19 in the combined dataset; subsequent rows show expression by brain region. Dots indicate the median value of the population. Absence of a violin plot in a row indicates that none or fewer than three cells from that particular brain region were mapped to the corresponding neuronal subtype. Black and red lines indicate consistent low and high expression of PCDH19 across areas, respectively; asterisks highlight clusters with marked variation in PCDH19 expression across cortical regions. CgG, Anterior cingulate gyrus; M1lm, primary motor cortex, lower limb region; M1ul primary motor cortex, upper limb region, A1C, primary auditory cortex. Download Figure 2-2, TIF file. [file enu-eN-NWR-0510-20-s05.tif]

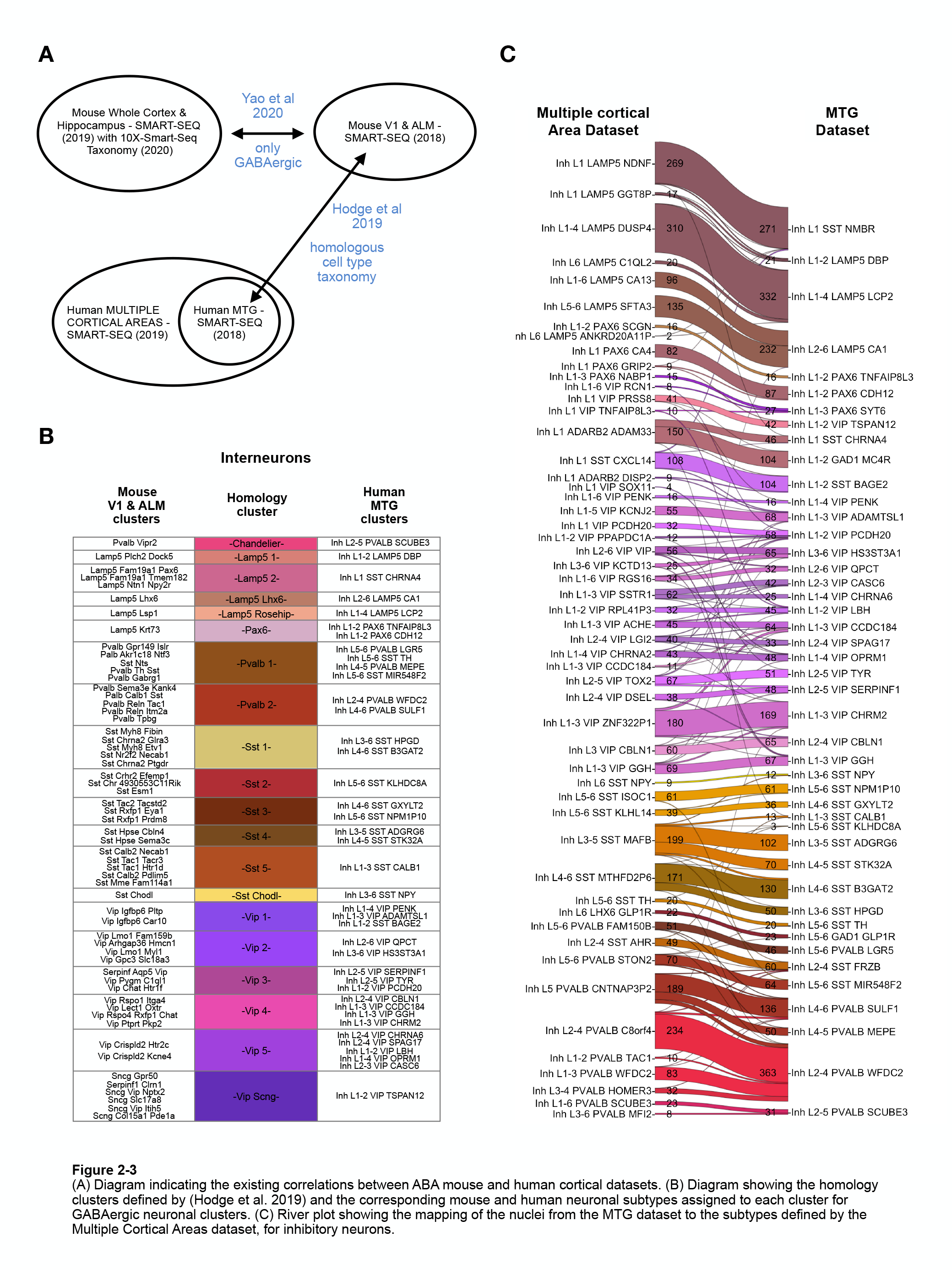

Supplement: Figure 2-3 — A, Diagram indicating the existing correlations between ABA mouse and human cortical datasets. B, Diagram showing the homology clusters defined by Hodge et al. (2019) and the corresponding mouse and human neuronal subtypes assigned to each cluster for GABAergic neuronal clusters. C, River plot showing the mapping of the nuclei from the MTG dataset to the subtypes defined by the Multiple Cortical Areas dataset, for inhibitory neurons. Download Figure 2-3, TIF file. [file enu-eN-NWR-0510-20-s06.tif]

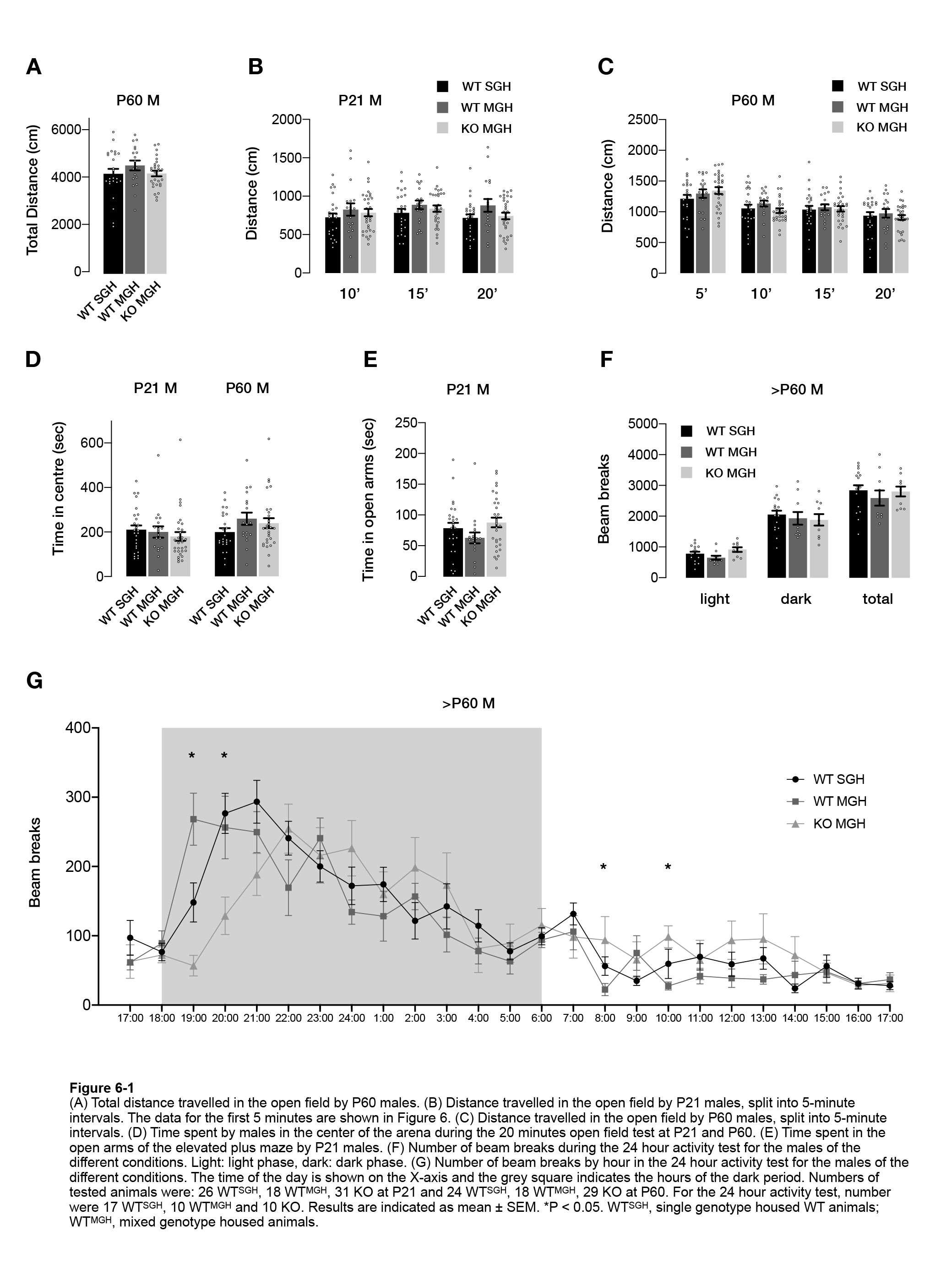

Supplement: Figure 6-1 — A, Total distance travelled in the open field test by P60 males. B, Distance travelled in the open field test by P21 males, split into 5 min intervals. The data for the first 5 min are shown in Figure 6. C, Distance travelled in the open field test by P60 males, split into 5 min intervals. D, Time spent by males in the center of the arena during the 20 min open field test at P21 and P60. E, Time spent in the open arms of the elevated plus maze by P21 males. F, Number of beam breaks during the 24 h activity test for the males of the different conditions. Light, light phase; dark, dark phase. G, Number of beam breaks by hour in the 24 h activity test for the males of the different conditions. The time of the day is shown on the x-axis, and the gray square indicates the hours of the dark period. The numbers of tested animals were as follows: at P21: WTSGH, 26; WTMGH, 18; KO, 31; at P60: WTSGH, 24; WTMGH, 18; KO, 29. For the 24 h activity test, the numbers were as follows: WTSGH, 17; WTMGH, 10; KO, 10. Results are indicated as the mean ± SEM. *p < 0.05. Download Figure 6-1, TIF file. [file enu-eN-NWR-0510-20-s07.tif]

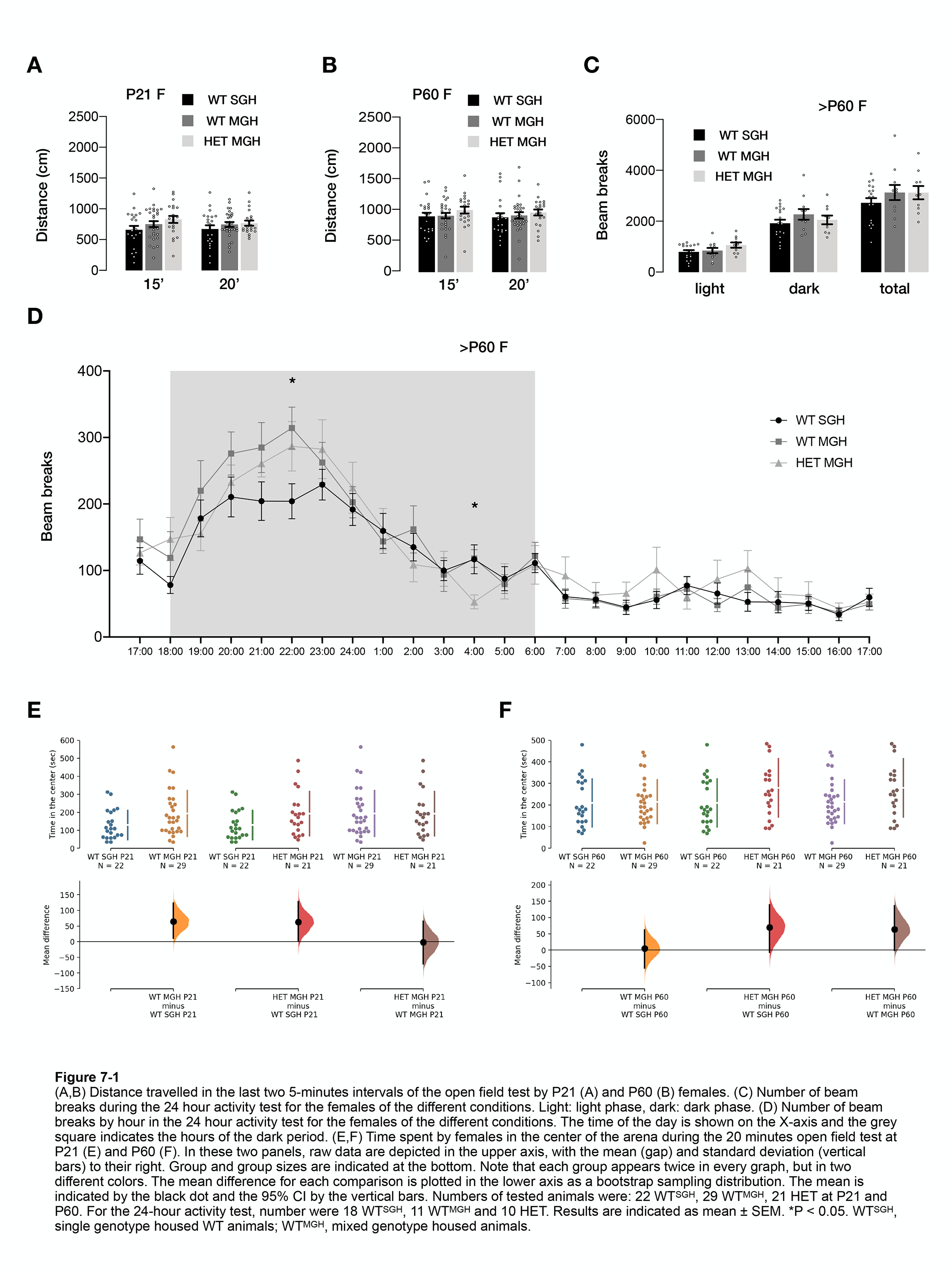

Supplement: Figure 7-1 — A, B, Distance travelled in the last two 5 min intervals of the open field test by P21 (A) and P60 (B) females. C, Number of beam breaks during the 24 h activity test for the females of the different conditions. Light, Light phase; dark, dark phase. D, Number of beam breaks by hour in the 24 h activity test for the females of the different conditions. The time of the day is shown on the x-axis, and the gray square indicates the hours of the dark period. E, F, Time spent by females in the center of the arena during the 20 min open field test at P21 (E) and P60 (F). In these two panels, raw data are depicted in the top axis, with the mean (gap) and SD (vertical bars) to their right. Group and group sizes are indicated at the bottom. Note that each group appears twice in every graph, but in two different colors. The mean difference for each comparison is plotted in the lower axis as a bootstrap sampling distribution. The mean is indicated by the black dot, and the 95% CI by the vertical bars. The numbers of tested animals were as follows: at P21 and P60: WTSGH, 22; WTMGH, 29; HET, 21. For the 24 h activity test, numbers were as follows: WTSGH, 18; WTMGH, 11; HET, 10. Results are indicated as the mean ± SEM. *p < 0.05. Download Figure 7-1, TIF file. [file enu-eN-NWR-0510-20-s08.tif]
